# Genome‐Wide CRISPRi Screening of Key Genes for Recombinant Protein Expression in *Bacillus Subtilis*

**DOI:** 10.1002/advs.202404313

**Published:** 2024-07-01

**Authors:** Xuyang Zhu, Hui Luo, Xinrui Yu, Huihui Lv, Lingqia Su, Kang Zhang, Jing Wu

**Affiliations:** ^1^ School of Biotechnology and Key Laboratory of Industrial Biotechnology Ministry of Education State Key Laboratory of Food Science and Resources International Joint Laboratory on Food Safety Jiangnan University Wuxi 214122 China

**Keywords:** Bacillus subtilis, gene regulation, genome‐wide CRISPRi screening, recombinant protein expression

## Abstract

*Bacillus subtilis* is an industrially important microorganism that is often used as a microbial cell factory for the production of recombinant proteins due to its food safety, rapid growth, and powerful secretory capacity. However, the lack of data on functional genes related to recombinant protein production has hindered the further development of *B. subtilis* cell factories. Here, a strategy combining genome‐wide CRISPRi screening and targeted CRISPRa activation to enhance recombinant protein expression is proposed. First, a CRISPRi library covering a total of 4225 coding genes (99.7%) in the *B. subtilis* genome and built the corresponding high‐throughput screening methods is constructed. Twelve key genes for recombinant protein expression are identified, including targets without relevant functional annotations. Meanwhile, the transcription of recombinant protein genes by CRISPRa is up‐regulated. These screened or selected genes can be easily applied to metabolic engineering by constructing sgRNA arrays. The relationship between differential pathways and recombinant protein expression in engineered strains by transcriptome analysis is also revealed. High‐density fermentation and generalisability validation results prove the reliability of the strategy. This method can be extended to other industrial hosts to support functional gene annotation and the design of novel cell factories.

## Graphical abstract

Based on genome‐wide CRISPRi screening, 12 repressor genes favorable for recombinant protein production are identified. sgRNA arrays are constructed to combine these novel targets with a CRISPRa‐driven activation strategy to achieve comprehensive multi‐gene regulation. The high‐yielding strains that are obtained have improved the yield of a variety of recombinant proteins.

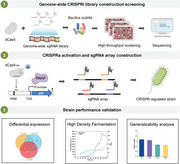

## 1 Introduction

The production of recombinant proteins in microbial cell factories has become a US$10 billion industry, with a wide range of products used in the pharmaceutical, food, and chemical industries.^[^ ^1^ ^]^ However, the intensive synthesis of recombinant proteins often leads to an imbalance in the allocation of metabolic resources between strain growth and protein production processes.^[^ ^2^ ^]^ Furthermore, overexpression of heterologous recombinant proteins is often limited by the poor environmental adaptability of the host, resulting in massive protein aggregation, misfolding, and upregulation of cellular stress levels.^[^ ^3^ ^]^ The complexity of the recombinant protein synthesis pathway and our lack of knowledge of the underlying regulation mechanism is a major reason why it is difficult to break the bottleneck of further improving recombinant protein expression in microbial cell factories.^[^ ^4^ ^]^

Currently, over 60% of industrial enzymes are produced in *Bacillus*.^[^ ^5^ ^]^ *Bacillus subtilis* is an important *Bacillus* model strain and its advantages, such as high secretion capacity and nonendotoxin production, have led to its development as an excellent host for recombinant protein expression.^[^ ^6^ ^]^ Over the years, many efforts have been made to improve recombinant protein expression in *B. subtilis*, including systematic studies of specific pathways^[^ ^7^ ^]^ and various types of omics analysis,^[^ ^8^ ^]^ and the modification targets identified so far are mainly involved in processes such as peptide translation, protein folding, secretion and protease degradation. In many cases, modification strategies for these targets can enhance the production of recombinant proteins,^[^ ^9^ ^]^ Chen increased AmyS and AmyL production through the enhancement of the Sec secretory pathway.^[^ ^10^ ^]^ In another study, DPEase activity was increased up to 6.12‐fold by the fusion expression of 4 native chaperones (DnaK, DnaJ, GrpE, and PrsA) in *B. subtilis*.^[^ ^11^ ^]^ However, there are limitations in the available targets for different types of recombinant protein. Additionally, the abundance of many factors that have been shown to play a facilitating role in the expression of recombinant proteins is not well represented in the results of omics analysis or existing gene annotations.^[^ ^12^ ^]^ As a result, it is difficult for rational approaches to identify these functional genes. Therefore, the directed evolution strategy based on library screening is expected to become an effective method for elucidating the synthesis mechanism of recombinant proteins and identifying the key modification targets.

The development of CRISPR‐based gene editing regulatory tools provides a simple and reliable solution for identifying key modification genes and elucidating gene‐phenotype associations. CRISPR interference (CRISPRi) can prevent the binding of RNA polymerase through the spatial site‐blocking created by the complex formed by dCas9 protein (deactivated Cas9, obtained by the D10A and H840A mutations) and single guide RNA (sgRNA) binding to DNA.^[^ ^13^ ^]^ Compared to previous functional gene identification methods, such as transposon mutagenesis^[^ ^14^ ^]^ or physicochemical mutagenesis^[^ ^15^ ^]^ library screening, CRISPRi‐based screening methods have the advantages of high library quality, strong targeting, and easy traceability. The easy design of sgRNA library allows it to be readily applied to high‐throughput screening of key genes in specific pathways.^[^ ^16^ ^]^ At the same time, the inducibility of the degree of gene expression repression by CRISPRi allows the characterization of the role of essential genes that determine the survival of bacteria in other pathways,^[^ ^17^ ^]^ increasing the library enrichment. In *B. subtilis*, this tool has been used to screen for drug targets and to characterize the contribution of essential genes to cell viability.^[^ ^13^ ^]^ Recently, CRISPRi based screening methods have been applied to identify key targets for increasing metabolic product or protein production in *Escherichia coli* ^[^ ^18^ ^]^ and *Corynebacterium glutamicum*.^[^ ^19^ ^]^ This demonstrates the promise of CRISPRi screens in the development of microbial cell factories. However, no relevant studies have been conducted on *B. subtilis*.

Here, we established a genome‐wide CRISPRi screening system in *B. subtilis* to investigate key genes that affect the expression of recombinant proteins. By targeting 4225 (99.7%) different open reading frames in the genome, the scope of our screen covered almost all genes encoded by *B. subtilis*. In addition, we established a stable CRISPR a/i system in the industrial strain WS9^[^ ^20^ ^]^ to overlay the suppression targets obtained from the screen, and targeted activation of the plasmid promoter, which further increased the yield of recombinant proteins. Finally, we analyzed the transcriptome differences of the final protein‐producing dominant strains. Our results not only provide a simple and feasible modification strategy for *B. subtilis* as a cell factory to express recombinant proteins but also improve our understanding of the recombinant protein production mechanism in *B. subtilis*, which can serve as a reference for other industrial strains used to express proteins.

## 2 Results

### 2.1 Regulation of *B. subtilis* 1A976 Gene Expression by CRISPRi System

**Figure 1:**
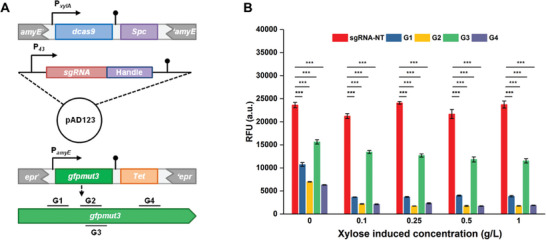
Construction and validation of the CRISPRi system in *B. subtilis* 1A976. A: Expression of CRISPRi elements, dCas9 and sgRNA is expressed at the *amyE* site and pAD123 plasmid, respectively. G1‐G4 are 4 sgRNAs targeting different positions of GFP. The distances from the starting codon are 40, 169, 172, and 263 nt, respectively. B: Relationship between target position and fluorescence inhibition at different xylose‐inducing concentrations. Values and error bars reflect the mean ± s.d. of 3 independent biological replicates (*n* = 3). Differences between multiple sets of data were compared using one‐way ANOVA followed by Tukey's test. ^*^ *p* < 0.05, ^**^ *p* < 0.01, and ^***^ *p* < 0.001.

We constructed a CRISPRi system in *B. subtilis* 1A976 with the P*_xyl_*‐*dcas9* cassette integrated into the genomic *amyE* locus and the P*_43_*‐*sgRNA* cassette on an *E. coli*‐*B. subtilis* shuttle plasmid pAD123, respectively. To investigate the ability of CRISPRi to repress gene transcription, we introduced GFP expression cassette into the genomic *epr* locus and 4 pAD123 plasmids containing specific sgRNAs targeting different positions of GFP, including 3 nontemplate strand targeted sgRNAs (G1:40, G2:169, G4:263) and one template strand targeted sgRNA (G3:172) (**Figure** **1**A). The numbers represent the distance from the target location to the starting codon. CRISPRi‐mediated gene interference achieved a 20‐fold downregulation of fluorescence levels under full induction (1% xylose induction) and was also able to cause a 3‐fold downregulation of fluorescence levels in the absence of inducers relying solely on promoter leakage expression (Figure 1B). sgRNA inhibition results for 4 different targets indicated that in *B. subtilis*, dCas9 interference with gene expression was significantly greater when targeting the non‐template strand than when targeting the template strand. In addition, when targeting the non‐template strand, the repression effect of CRISPRi in *B. subtilis* did not depend on the distance of the dCas9 binding site from the start codon. Thus, our CRISPRi tool can be used to effectively regulate gene expression levels in *B. subtilis* and can be further exploited for functional gene discovery.

### 2.2 Genome‐Wide CRISPRi Screening for Key Targets Involved in Recombinant Protein Expression

To investigate the application of our CRISPRi screening method in the expression of recombinant proteins, we selected *Bacillus circulans* β‐galactosidase (β‐Gal) and *Pyrococcus furiosus* hyperthermophilic amylase (Pfa) as intracellular and extracellular model proteins, respectively. Then, we constructed an initial CRISPRi library containing 6000 specific sgRNAs which targeted 4225 different ORFs (99.7% genome ORF coverage) based on the sequence information of *B. subtilis* 168 collected from SEED view. The NGS (Next‐generation sequencing) assay results showed high homogeneity and sequence accuracy of the synthesized sgRNA libraries (Figure S1, Supporting Information). Then these library plasmids were transferred into the strains encoding the protein of interest genes and *dcas9* to generate the CRISPRi screening libraries. The non‐targeted sgRNA was used as a control. To ensure the diversity and stability of the library, all screening procedures were performed without the addition of xylose induction to avoid excessive suppression of essential genes leading to the loss of this phenotype in the library or the deviation caused by the addition of inducers.

**Figure 2:**
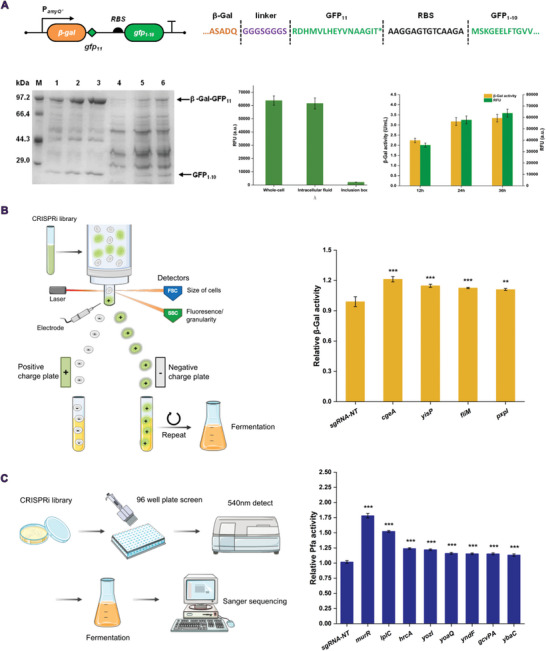
CRISPRi library screening workflow. A) Construction of split‐GFP sensor and validation of split‐GFP sensitivity by β‐Gal enzyme activity versus fluorescence assay. The 2 components of the sensor were expressed in tandem by the addition of RBS. The electrophoretic bands represent the amount of soluble β‐Gal‐GFP_11_, GFP_1‐10,_ and inclusion bodies in the intracellular fluid and wall‐breaking precipitates at different sampling times. M stands for Marker, 1: 12 h intracellular fluid; 2: 24 h intracellular fluid; 3: 36 h intracellular fluid; 4: 12 h inclusion body; 5: 24 h inclusion body; 6: 36 h inclusion body. B) β‐Gal flow sorting workflow and identification of screening factors. C) Pfa high‐throughput screening workflow and screening factor identification. Relative enzyme activity represents the ratio of enzyme activity between single gene‐inhibited strains and non‐targeted sgRNA‐expressing strains (control group). Values and error bars reflect the mean ± s.d. of 3 independent biological replicates (*n* = 3). Differences between multiple sets of data were compared using one‐way ANOVA followed by Tukey's test. ^*^ *p* < 0.05, ^**^ *p* < 0.01, and ^***^ *p* < 0.001.

For β‐Gal, we constructed a split‐GFP sensor to improve screening throughput.^[^ ^21^ ^]^ GFP_11_ and GFP_1‐10_ do not fluoresce when present alone, while they give a fluorescent signal when both are present in the system. The GFP_11_ tag is attached to the C‐terminus of β‐Gal using an eight amino acids flexible linker. To homogenize the expression levels of β‐Gal‐GFP_11_ and GFP_1‐10_, we added an RBS to concatenate GFP_1‐10_ to the downstream of β‐Gal‐GFP_11_, both components of proteins are regulated by P*_amyQ'_*. Determination of bacterial protein expression levels and fluorescence at different fermentation times showed that split‐GFP was able to respond to the changes in protein expression levels. By further evaluating the fluorescence levels of different cell components, we found that only soluble proteins could be detected for fluorescence, while misfolded inclusion bodies did not show any fluorescence signals (**Figure** **2**A). These results indicate that the split‐GFP sensor is sensitive and feasible for tracing β‐Gal soluble expression levels. CRISPRi libraries were obtained by transforming sgRNA plasmid libraries into the chassis strain *B. subtilis* 1A976D with β‐Gal‐GFP_11_ and GFP_1‐10_ expression plasmid. The cultured library bacteria were subjected to several rounds of fluorescence‐activated cell sorting. In the first round, a total of 69 835 cells were analyzed and 3500 cells with the top 5% fluorescence value were collected. After enrichment and cultivation, a total of 559 cells with the top 1% fluorescence value were selected in the second round for re‐screening validation (Figure S2, Supporting Information). Flow cytometry analysis was performed on the enriched cells, and after 2 rounds of targeted enrichment, the fluorescence value of the cell population increased to 1.71 times that of the original library strain, indicating that individuals with high expression levels were effectively enriched. The bacterial cells collected by flow cytometry were resuscitated on a plate and then cultured in 96 well plates. Finally, individuals with high fluorescence intensity were verified at the shake flask level for β‐Gal expression. Four strains increased the production of β‐Gal by 20.4%, 14.5%, 12.2%, and 11.1% after 36 h shake flask fermentation (Figure 2B). By sequencing the spacer sequences, these 4 target genes were identified as *cgeA*, *yisP*, *fliM* and *pxpI*. Among them, *cgeA* is related to spore formation, *yisP* is a farnesyl diphosphate phosphatase, *fliM* is involved in flagellar movement and *pxpI* is a putative proline cyclase. This suggests that the synthesis of intracellular recombinant proteins is influenced by several cellular processes.

For Pfa, A total of 6160 colonies were screened in 96 well plates. The CRISPRi‐library strains were incubated at 33 °C for 60 h according to the method described above and then assayed for supernatant enzyme activity. The high‐activity strains identified in the 96‐well plate were re‐screened at the shake flask level and sgRNA sequencing was performed on individuals with increased enzyme activity. Inhibition of eight targets was found to have a positive effect on the expression of Pfa. Inhibition of *murR*, *lplC*, *hrcA*, *gcvPA*, *ybaC*, *yozI*, *yoaQ*, and *yndF* increased Pfa enzyme activity by 77.4%, 52.1%, 23.0%, 14.6%, 13.9%, 22.8%, 16.5% and 15.1% respectively (Figure 2C). HrcA is a known transcriptional repressor of chaperone proteins. It has been reported that the knockdown of *hrcA* in *B. subtilis* can enhance the production of recombinant protein,^[^ ^22^ ^]^ indicating a high level of confidence in the results of our screening. The remaining targets are mainly related to peptidoglycan recycling (*murR*), amino acid metabolism (*gcvPA*) and membrane transport (*lplC*), spore production (*yndF*). We also detected some targets without gene annotation (*ybaC*, *yozI*, *yoaQ*). Similar results were observed in the screening process of β‐Gal, indicating that a large number of factors not directly involved in protein synthesis have an effect on the efficient expression of recombinant proteins. On the one hand, the gene annotation related to the protein synthesis pathway of *B. subtilis* is still incomplete. On the other hand, suppression of metabolism‐related genes may have led to a reduction in metabolic flux through the relevant pathways, further regulating the allocation of metabolic resources. However, the specific mechanisms of this regulation still need further explanation. The key genes affecting the expression of different types of recombinant proteins are highly variable, which may be the reason why no factors with universal properties have been found. During the screening process, the screening efficiency of Pfa as a model protein was significantly higher than that of the intracellular model protein β‐Gal. This may be due to the characteristics of *B. subtilis* as a secretory expression host. Therefore, in the subsequent transformation, we continued our study using Pfa as a model protein.

### 2.3 CRISPR a/i Construction and Screening Target Integration in Industrial Strain WS9

Compared to the model strain *B. subtilis* 168 and its derivatives, *B. subtilis* WS9, with 6 extracellular proteases knocked out, has a higher secretion capacity and is more suitable for high‐density fermentation.^[^ ^20^ ^]^ For industrial application considerations, we tend to integrate the screened targets into the WS9 strain overexpressing *comK* (WS9C) with optimized gene editing efficiency to further develop its potential as an industrial strain.

**Figure 3:**
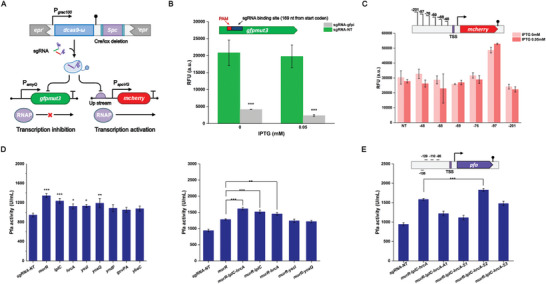
CRISPRa activation characterization and sgRNA array construction. A) Working principle of dCas9‐ω multigene regulation. B) Characterization of the ability of dCas9‐ω to inhibit fluorescence in WS9C. The sgRNA used to test the ability of dCas9‐ω targeted the 169 nt downstream of the *gfp* start codon. C) Activation of mCherry fluorescence at different distances upstream of TSS by dCas9‐ω targeting. The sgRNAs used for activation were targeted upstream of the P*_spoVG_* promoter TSS at 48, 68, 69, 76, 97, 201 nt respectively. D,E) Effect of sgRNA arrays on the expression of Pfa. All shake flask fermentations were induced with 0.05 mM IPTG. Values and error bars reflect the mean ± s.d. of 3 independent biological replicates (*n* = 3). Differences between multiple sets of data were compared using one‐way ANOVA followed by Tukey's test. ^*^ *p* < 0.05, ^**^ *p* < 0.01, and ^***^ *p* < 0.001.

We constructed a dual‐functional CRISPR a/i system^[^ ^23^ ^]^ in WS9C rather than a single CRISPRi system, which activates and inhibits transcription through designing locus‐specific sgRNAs and increases the convenience of strain modification (**Figure** **3**A). The ω subunit of RNA polymerase was connected to the C‐terminal of dCas9 to construct dCas9‐ω,^[^ ^24^ ^]^ which was regulated by the IPTG (Isopropyl‐β‐D‐thiogalactopyranoside)‐inducible promoter P*_grac100_*.^[^ ^25^ ^]^ The P*_grac100_*‐*dcas9‐ω* expression cassette was integrated into the *epr* locus (one of the WS9 knockout proteases). Meanwhile, we introduced 2 expression cassettes, P*_amyQ_*‐*gfp* and P*_spoVG_*‐*mcherry*, into the *nprE* locus to validate the regulatory ability of dCas9‐ω. To improve the genetic stability of the strain, sgRNA expression cassettes were integrated into the *lacA* locus instead of expressing by plasmid. The sgRNA targeting 169 nt downstream of the GFP initiation codon was used to investigate the inhibition ability of dCas9‐ω. After culturing the recombinant bacteria for 24 h, the fluorescence value was determined. We found that P*_grac100_* also showed leaky expression. The GFP fluorescence value decreased by 5.02 times without the addition of IPTG and 8.36 times under the induction of 0.05 mM IPTG (Figure 3B). For the research on the activation ability of dCas9‐ω, the GC‐rich promoter P*_spoVG_* is beneficial for the design of different targeted sgRNAs, because GC‐rich promoters are likely to have PAM sequence (NGG). Six sgRNAs targeting TSS upstream of P*_spoVG_* at different distances (48, 68, 69, 76, 97, 201 nt) were introduced into the *lacA* locus and only the sgRNA targeting 97 bases upstream of the P*_spoVG_* TSS could demonstrate effective activation at both IPTG inducer concentrations of 0 and 0.05 mM, increasing the mCherry fluorescence levels to 1.60 and 1.90 times, respectively (Figure 3C). This indicates that the CRISPR a/i system can effectively regulate the transcription of multiple genes in WS9C.

We investigated the effect of down‐regulating the eight genes identified in the above CRISPRi screen on Pfa expression in the *dcas9‐ω* encoding strain WS9D. All sgRNA expression cassettes were individually integrated into the *lacA* locus, and recombinant bacteria were cultured at 33 °C under 0.05 mM IPTG‐inducing conditions for 60 h. Enzyme activity assays revealed that inhibition of all eight targets within WS9D resulted in an up‐regulation of Pfa expression. These results suggest that the targets obtained from the screen in the model strain have high generality to WS9D. We then reconstructed the sgRNA arrays by combining other candidate sgRNAs with the sgRNA targeting *murR*, which showed the most significant improvement in Pfa production, and constructed 4 dual‐targeted repressor strains (*murR*‐*lplC*, *murR*‐*yoaQ*, *murR*‐*yozI*, and *murR*‐*hrcA*). The repression combinations of *murR* and *lplC* increased the enzyme activities of Pfa by 61.7% and the inhibition combination of *murR* with *hrcA* increased the enzyme activity of Pfa by 54.8%. None of the other dual‐target candidates showed superior effects to the single‐target inhibition of *murR* (36.7%). In this case, we further constructed a *murR*‐*lplC*‐*hrcA* triple‐target inhibitor strain. The *hrcA*‐targeted sgRNA expression cassette was integrated into the *mpr* locus, which was different from the sgRNAs array (*lacA* site) of *murR*‐*lplC*, to avoid introducing too many repetitive sequences within the same locus and causing genetic instability. The triple‐target inhibitor strain increased Pfa expression by 71.6% under the same fermentation conditions (Figure 3D). The roles and mechanisms of these targets in the recombinant protein expression process are discussed in the subsequent chapters.

### 2.4 CRISPRa Mediated Promoter Activation Increases Recombinant Expression

Similar to CRISPRi, CRISPRa regulates transcriptional activity by targeting sgRNAs to specific sites. However, CRISPRa has very strict requirements for the distance between the sgRNA target site and the TSS of the promotor.^[^ ^26^ ^]^ The mCherry fluorescence activation results we previously observed also confirm this point. On the other hand, the advantages of CRISPRa are obvious, in contrast to the traditional method of overexpressing genes by massively increasing the copy number, there is no need to introduce expression frames other than dCas9‐ω and sgRNA. Therefore, we chose to search for suitable activation targets on the high GC content and high transcriptional intensity promoter P*_amyQ'_*, which regulates the POI (protein of interest) on the plasmid, to further improve the transcriptional activity of pfa.

Four sgRNAs targeting different distances upstream of the TSS were integrated into the genome of the *murR*‐*lplC*‐*hrcA* triple‐target inhibitor strain, specifically binding 135 bp (anti‐sense strand, A1), 129 bp (sense strand, S1), 110 bp (sense strand, S2) and 95 bp (sense strand, S3) upstream of the TSS. The activated targeted sgRNAs expression cassette were introduced to the *mpr* locus and formed an array with *hrcA* targeted sgRNA. After cultivation, we measured Pfa enzyme activity and found that only S2 could further enhance Pfa expression. The enzyme activity increased by 93.8% at the 60 h shake flask level, reaching 1827.8 U mL^−1^ (Figure 3E). The remaining sgRNAs candidates showed varying degrees of decreased expression levels, which may be due to transcriptional inhibition caused by improper targeting. We successfully achieved both activation and inhibition in the engineered strain WS9D by constructing simple sgRNAs arrays and increased the production of recombinant proteins. The CRISPR regulated strain targeting 4 sites (*murR*, *lplC*, *hrcA* were inhibited and *pfa* was activated) is named WS9MLHS.

### 2.5 Investigation of CRISPR‐Engineered Bacteria in a 3L Fermenter

**Figure 4:**
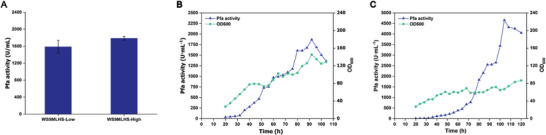
WS9MLHS High‐Density Fermentation Validation. A: Yield of Pfa shake flask fermentation at different IPTG induction concentrations for 60 h. IPTG induction was 0 mM (Low) and 0.05 mM (High), respectively. B‐C: WS9MLHS production of Pfa in a 3L fermenter, B shows the group induced by the addition of 0.05 mM IPTG, and C shows the group without the addition of an inducer. Values and error bars reflect the mean ± s.d. of 3 independent biological replicates (*n* = 3).

A simplified fermentation process is more favorable for high‐density fermentation cultures. To evaluate the need for IPTG in high‐density fermentation processes, we first investigated the dependence of CRISPR regulation on IPTG in WS9MLHS. The enzyme activity of the low induction group (Low) reached 1587.7 U mL^−1^ after 60 h of fermentation in shake flasks, which was only 12.6% lower than that of the IPTG‐induced group (High) (**Figure** **4**A). This indicated that the P*_grac100_* promoter‐mediated expression of dCas9‐ω is not dependent on IPTG inducers, and its leaked expression level can basically meet the regulatory requirements, which is favorable for the control of the fermentation process as there is no need to consider additional induction steps. We verified the ability of WS9MLHS to express recombinant proteins under high‐density fermentation conditions, which were divided into 2 groups, IPTG‐induced (0.05 mM) (High) and uninduced (Low). The growth of the High group was significantly better than that of the Low group during the 120 h fermentation, but the highest enzyme activity of the High group reached a maximum of only 1865.4 U mL^−1^ after 92 h of fermentation (Figure 4B), whereas the highest enzyme activity of the Low group reached a maximum of 4653.2 U mL^−1^ after 102 h, which was a 2.93‐fold increase compared to the shake flask and was the highest level reported to date (Figure 4C). This suggests that the addition of IPTG is detrimental to the enzyme production of WS9MLHS during high‐density fermentation.

### 2.6 CRISPR‐Regulated Bacterial Strain Transcriptome Analysis

**Figure 5:**
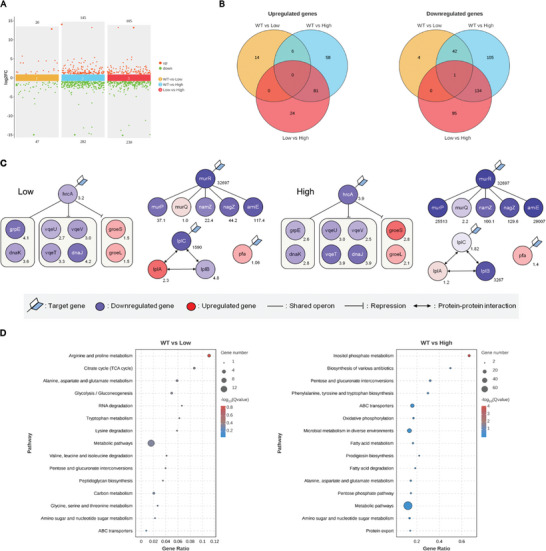
WS9MLHS transcriptional differential analysis. A) WT versus Low versus High multi‐group difference analysis. Points at the top of the graph are up‐regulated expressed genes in the comparison group, and at the bottom are down‐regulated expressed genes. B) Venn diagnosis of differences between groups. Sets that overlap represent the number of genes shared between groups, and those that do not overlap represent genes unique to each comparison group. C) Changes in abundance of CRISPR‐targeted genes. Numbers represent fold change in FPKM compared to the WT group, blue represents transcriptional down‐regulation, and red represents transcriptional up‐regulation. D) Differential KEGG number statistics. The top 15 Q‐value categories were selected for listing.

In order to study global changes in WS9MLHS, we performed transcriptome analysis of WT, WS9MLHS‐Low without inducers and WS9MLHS‐High with IPTG inducers added, and a total of 3795 transcripts were detected, covering 93.77% of the coding genes of *B. subtilis* (Figure S3, Supporting Information). A total of 564 genes were significantly altered (FDR<0.05 and |log2FC|>1) when comparing the 3 data sets, WT versus Low, WT versus High, and Low versus High, of which 183 genes were significantly up‐regulated and 381 genes were significantly down‐regulated. Only 67 differentially expressed transcripts, or 1.8% of the total transcripts, were detected in the Low group without inducer compared to the WT control, whereas 427 transcripts, or 11.5% of the total transcripts, were significantly altered in the High group with inducer compared to the WT, suggesting that the IPTG inducer has a significant effect on the global transcription of the *B. subtilis* strain (**Figure** **5**A,B). Varying degrees of corresponding abundance changes were observed in the target sites of the CRISPR a/i regulatory strains, and the changes in transcript abundance of the related operons, upstream and downstream regulatory genes, and intercalating factors are shown in the figure (Figure 5C). The transcript abundance of *murR* was significantly suppressed in both experimental groups, bringing down the transcription of genes within its operon together. The repression of downstream genes was more pronounced in the High group. In contrast, the level of *lplC* repression in the low group was much higher than that in the high group. *lplC* needs to interact with *lplB* and *lplA* to function as a transporter protein, and different levels of *lplC* repression up‐regulated *lplA* transcription by 2.3‐fold and 1.2‐fold, respectively. The level of up‐regulation was proportional to the level of repression, and it was suggested that *lplA* may act as a backfill mechanism within the operon. Repression of *hrcA* increased the transcriptional abundance of the intracellular chaperone factor GroES/GroEL.^[^ ^22^ ^]^ This facilitated improved folding of the recombinant protein before it was secreted out of the cell membrane.^[^ ^27^ ^]^ The transcriptional abundance of pfa showed different degrees of up‐regulation as well. These data indicate that CRISPR regulation in *B. subtilis* is precise.

We examined the genes with the most significant differential changes, and both the Low and High groups significantly down‐regulated the transcription of the MurNAc recycling operon, caused by the suppression of *murR*, primarily reducing the recycling of extracellular peptidoglycan. *B. subtilis* does not rely on cell wall recycling like gram‐negative bacteria, and it has been shown that Δ*murQ* mutants do not have growth defects in complete media.^[^ ^28^ ^]^ The *Xpf* regulon, which regulates PBSX (a phage‐like bacteriocin of *B. subtilis*) cell autolysis,^[^ ^29^ ^]^ was also downregulated in the Low group, and we found a similar phenomenon in the differential gene table of the High group, which is beneficial for maintaining stable strain growth. In addition, the High group showed a decrease in the abundance of the *acoR* regulon and an increase in the *epsA* regulon compared to the Low group, which regulate acetylene use and extracellular polysaccharide synthesis, respectively (Figure S4A, Supporting Information). KEGG enrichment analysis of the DEGs revealed enrichment in terms of “ABC transporters”, “RNA degradative”, and various amino acid metabolism (Figure 5D). A number of ABC transporter proteins were inhibited, mainly involving zinc, manganese, iron (II), molybdate, and biotin transport utilization, and the enhancement of recombinant protein expression by inhibition of ABC transporter proteins has been shown to be effective in other studies.^[^ ^19^ ^]^ GO analysis also showed changes in the strain's transporter capacity, which is in line with our previous expectations and may be related to changes in cell membrane properties (Figure S4B, Supporting Information). Enrichment of the term “RNA degradative” is associated with the inhibition of *hrcA*, and down‐regulation of *hrcA* activates the expression of the intracellular chaperone protein GroES/GroEL, which in turn improves intracellular folding conditions and optimizes intracellular quality control processes. The enrichment of “Alanine, aspartate, and glutamate metabolism” is consistent with the pattern of transcriptional differences in the production of recombinant proteins by yeast.^[^ ^30^ ^]^ We also observed a perturbation of inositol metabolism, lipid metabolism, and some cofactor metabolism by IPTG induction.

### 2.7 Generalisation of WS9MLHS as an Expression Host for Recombinant Proteins

**Figure 6:**
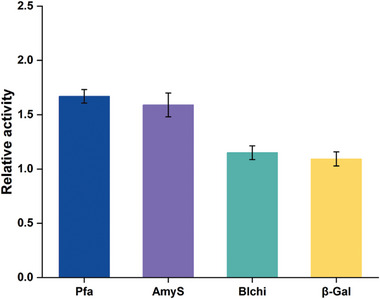
Generalisability of WS9MLHS for expression of different recombinant proteins. The relative activity represents the ratio of the enzyme activity of the recombinant protein expressed in WS9MLHS to the enzyme activity expressed in WS9D. Values and error bars reflect the mean ± s.d. of 3 independent biological replicates (*n* = 3).

In previous studies, we have found that different recombinant proteins require different regulatory targets. Even for the same recombinant protein, combinations of target modifications are not always effective. It has been reported that there are similarities in the transcriptional correspondences of the high‐yielding strains even when different strain mutations or modifications have been performed.^[^ ^30^ ^]^ Therefore, compared to verifying the generality of a single factor, we focused on the high‐yielding strain WS9MLHS and verified its versatility in expressing different recombinant proteins. Three additional recombinant proteins were used to validate the generalisation of WS9MLHS, including *Geobacillus stearothermophilus*‐derived hyperthermic amylase (AmyS), *Bacillus licheniformis*‐derived chitinase (Blchi) and *B. circulans*‐derived β‐Gal as mentioned above. Fermentation data showed that WS9MLHS promoted the expression of all 3 recombinant proteins, increasing the expression of AmyS, Blchi, and β‐Gal by 59.3%, 15.9%, and 9.3%, respectively (**Figure** **6**). All fermentation processes were carried out without adding additional inducers. This suggests that our regulatory targeting is not product specific, but rather optimizes the synthesis processes of recombinant proteins.

## 3 Disscussion

CRISPRi‐mediated functional gene screening has shown promising potential for essential gene analysis, identification of metabolic nodes, and interpretation of expression‐fitness relationships between species.^[^ ^31^ ^]^ Here, we developed a genome‐wide CRISPRi screening method to identify key genes for recombinant protein production in *B. subtilis*. We also developed an adapted high‐throughput screening method. Our experiments revealed the positive impact of CRISPRi library screening on the discovery of functional genes for recombinant protein expression, and more importantly, the factors obtained from these screens can be genetically engineered by constructing sgRNA arrays, which can effectively enhance recombinant protein production. Our experiments demonstrate the high site selectivity of dCas9‐ω for CRISPRa activation. It also proves that combinatorial CRISPR regulation is precise and stable during both shake‐flask culture and high‐density fermentation. In addition, our experiment revealed the differential pathways in high‐yielding strains through transcriptome analysis. Finally, we also assessed the generality of the modified strain WS9MLHS.

The greatest advantage of CRISPRi‐based screening over previous gene inactivation screening methods such as ARTP mutagenesis or Tn‐seq^[^ ^32^ ^]^ is that CRISPRi has a more flexible level of gene suppression, the different DNA strand targeting allows a wider range of suppression levels.^[^ ^31a^ ^]^ This is crucial for the identification of housekeeping genes; excessive suppression or silencing due to knockdowns often leads to growth defects in the strain and thus loss of genotype, which further affects the enrichment of the library. Another advantage of CRISPRi screening is its high coverage. sgRNA design only needs to consider complementarity between the spacer sequence and the sgRNA handle^[^ ^33^ ^]^ as well as the PAM site, which is applicable to the vast majority of CDSs, including some short coding genes below 300 nt. The designability of sgRNA libraries allows flexibility and precision in targeting range. This distinguishes CRISPRi screening from Tn‐seq, mainly by greatly reducing the large amount of library redundancy and uneven homogeneity introduced by random transpositions. In this study, split‐GFP and 96‐well plate colorimetric methods were used to screen intracellular and extracellular model proteins, respectively, and we found that the split‐GFP‐based intracellular screening method was convenient to implement, but the screening throughput of extracellular model proteins was relatively low. Although microfluidic droplet‐based FACS screening methods have been developed,^[^ ^19^ ^]^ for industrial enzymes, high temperature or acidic reaction conditions are challenging for the application of microfluidics. Thanks to the low‐redundancy nature of CRISPRi libraries, 96‐well plate‐based screening has also yielded satisfactory results. However, for extracellular secretion products, there is still a need to develop high‐throughput screening methods that can accommodate different reaction conditions.

Functional gene data associated with recombinant protein expression has been difficult to accurately determine for 2 reasons. First, the degree of regulation of factors identified by irrational or rational methods can affect the yield of recombinant proteins.^[^ ^34^ ^]^ Second, these targets are often product‐specific, and modifications against them may not work for different target products.^[^ ^10^ ^]^ Here, we provide the library with a rich selection of targets, and each sgRNA in the library is unique. However, there are differences in key targets for specific recombinant proteins. For example, overexpression of GroES/GroEL, annotated as an intracellular chaperone protein, resulted in decreased and increased enzyme activities of AmyL and AmyS, respectively.^[^ ^10^ ^]^ Different recombinant proteins are likely to respond differently to specific factors, which can only be answered experimentally. We performed a genome‐wide CRISPRi screen for each of the different recombinant proteins to separately identify the factors involved, which is an acceptable cost given the maneuverability of the CRISPRi libraries.

Our results showed that CRISPRa activation is strictly site‐selective. Some studies have pointed out that there is a periodicity between the activation ability of CRISPRa and the target distance, and the RNAP recruitment ability of dcas‐polymerase subunit fusion protein is influenced by the direction of the DNA helix.^[^ ^35^ ^]^ Compared to the certainty of CRISPRi targeting sgRNA design, CRISPRa requires a much larger library capacity to include as many validated targets as possible, which will directly increase the noise in the screening process.^[^ ^36^ ^]^ The activation effect is also related to the transcription factor to which dCas9 is bound. Another reason that limits the ability of CRISPRa to perform CRISPRi‐like screening functions is that native promoters are difficult to activate because they tend to be tightly regulated.^[^ ^37^ ^]^ From a sgRNA design perspective, native promoters do not always have appropriate upstream PAM sequences available for binding, and for some short promoters, the sgRNA target may enter the coding region of the upstream CDS, preventing transcription. We have designed sgRNAs on both the template and non‐template strands to obtain suitable activation sites and have successfully identified effective activation sites. The use of dCas9 protein mutants with lower PAM preference or the design of easy‐to‐activate promoters instead of native promoters may be more favorable for the realization of CRISPRa.^[^ ^35^, ^38^ ^]^

Genetically engineered overlays of factors obtained by identification are not always effective, and our results showed that among the eight factors screened only the combination of *murR*, *lplC*, and *hrcA* showed better results than before the combination, suggesting that there are interactions between these components. The downregulation of multiple genes can lead to imbalances in cellular processes or metabolism that are not reflected when individual genes are suppressed. The complexity of gene regulation means that the effects of combinatorial strategies are difficult to predict and need to be validated experimentally. We have considered the possibility that homologous recombination of repetitive sequences may reduce genetic stability^[^ ^39^ ^]^ when constructing sgRNA arrays. Whether the classic dCas9 or dCas12a,^[^ ^40^ ^]^ which is often used in *B. subtilis*, the construction of sgRNA arrays inevitably introduces repetitive sequences, mainly promoters, sgRNA handles, and terminators that are paired with the spacer sequences. Therefore, we integrated the sgRNA arrays into the 2 knocked‐out protease locus separately during construction to avoid a large number of repetitive elements at the same locus. Relying on the computational design of expression elements for non‐repetitive sgRNAs can solve this problem, especially when the number of targets is large.^[^ ^41^ ^]^

KEGG analysis revealed enrichment of the pathways in which the inhibited targets are involved, including cell wall recycling regulated through the *murR* operon, ABC transport regulated through the *lplC* operon, and quality control induced by *hrcA* inhibition. Inhibition of ABC transport proteins has been shown to promote recombinant protein expression in a recent study,^[^ ^19^ ^]^ and the increased chaperone protein activity mediated by *hrcA* inhibition is also the main reason for further optimization of intracellular folding and quality control processes. However, no studies have yet shown that blocking the cell wall recycling process in strains has a positive effect on recombinant protein yield. As a gram‐positive bacterium, the growth of *B. subtilis* in nutrient‐rich media does not depend on this process,^[^ ^28^ ^]^ and we hypothesis that this pathway can be streamlined during fermentation, although the exact mechanism needs to be further investigated. Our experiments also observed active amino acid metabolism of alanine, aspartate, glutamate, arginine, and proline, and down‐regulation of PBSX lysis genes in both WS9MLHS groups. Arginine and proline have been shown to be favorable for the expression of human‐like collagen (HLC) II.^[^ ^42^ ^]^ In addition, L‐arginine has been reported to inhibit protein aggregation in *E. coli* and reduce the inclusion bodies.^[^ ^43^ ^]^ The enrichment of the term “Alanine, aspartate, and glutamate metabolism” was also observed in other high‐yielding strains mentioned in the enrichment analyses.^[^ ^30^ ^]^ The downregulation of PBSX autolysis genes will improve cellular homeostasis. The revelation of these pathways provides a lesson for subsequent rational modification of cell factories.

Another interesting finding was the expression pattern of dCas9‐ω. From the perspective of transcriptome differential analysis, the perturbation of gene transcription without the addition of the IPTG inducer was very small compared to that of the control group, suggesting that dCas9‐ω‐mediated regulation was mild and precise for the host. However, the addition of IPTG caused very large perturbations in the global transcription of the host bacterium, with differentially expressed genes accounting for 11.5% of total transcripts, and these effects were also demonstrated during high‐density fermentation. As inducible promoter elements are still not as abundant in *B. subtilis* as in *E. coli*, expression of dCas9‐ω using a suitable constitutive promoter may be a good alternative.^[^ ^40^ ^]^

## 4 Conclusion

In conclusion, we have proposed a combined strategy of genome‐wide CRISPRi screening and targeted activation of CRISPRa to increase the production of recombinant proteins in *B. subtilis* and have developed a corresponding high‐throughput screening method. By sequencing the sgRNAs of the sorted individuals, we identified 12 repressor genes that promote the production of different types of recombinant proteins, 11 of which were empty in protein expression‐related annotations. Increased recombinant protein expression was also observed when these genes were inhibited in the industrial strain WS9. We demonstrated that a CRISPRa‐based promoter activation strategy for recombinant proteins is feasible in *B. subtilis* and combines activated sgRNAs with repressed sgRNAs by constructing sgRNA arrays. The CRISPR‐regulated strain WS9MLHS, which simultaneously mediated the transcriptional down‐regulation of 3 genes, *murR*, *lplC*, and *hrcA*, and the transcriptional up‐regulation of *pfa*, showed a significant advantage in recombinant protein yield. In this study, we revealed the effectiveness of this strategy through differential gene analysis, high‐density fermentation, and evaluation of the generalizability of the expression of different recombinant proteins. In addition, the strategy can be easily applied to increase the production of other natural products or to guide the development of other microbial cell factories.

## 5 Experimental Section

### Materials

TIANprep Mini Plasmid Kit and TIANgel Purification Kit were purchased from Tiangen (Tianjin, China). 2 × Phanta Max Master Mix, ClonExpress Ultra One Step Cloning Kit and One‐Step PAGE Gel Fast Preparation Kit were acquired from Vazyme (Nanjing, China). Restricted enzymes were from Takara (Japan). Q5 High‐Fidelity DNA Polymerase was purchased from NEB (United States of America). Pjmp1 and pAD123^[^ ^44^ ^]^ plasmids were purchased from BIO SCI (Hangzhou, China). The pUB110^[^ ^45^ ^]^ and pET24a plasmids were stored in the laboratory. IPTG, ampicillin, tetracycline, kanamycin, spectinomycin, and chloramphenicol were purchased from Sangon Biotech (Shanghai, China). Xyltose, NaCl, phosphate, and glycerol were acquired from SINOPHARM (Shanghai, China). Peptone and yeast extract were purchased from OXOID (United States of America), and soybean peptone and yeast extract powder for 3L fermenter cultivation were purchased from Yuan Peptide Biotechnology Co. (Shanghai, China), LTD and Angel (Yichang, China), respectively.

### Plasmid Construction

The construction of the plasmid vector was carried out using standard molecular biology methods. For the expression plasmid pUB110‐POI in *B. subtilis*, as it did not have an *E. coli* replication origin, we used the POE‐PCR method^[^ ^46^ ^]^ to connect the recombinant fragments. The DNA fragments were amplified in vitro to form a polymer and then transformed into competent *B. subtilis* 1A976 cells.^[^ ^47^ ^]^ Subsequently, the correct clones were selected on a 30 µg mL^−1^ kanamycin‐resistant plate. For both the pAD123 sgRNA shuttle vector and the pET24a integration vector, one‐step cloning or multi‐fragment assembly was performed using the Gibson assembly method. The plasmid assembled in vitro was transformed into competent *E. coli* JM109 cells and selected on a 100 µg mL^−1^ ampicillin or 30 µg mL^−1^ kanamycin plate. The specific vector sequence information details can be found in the supporting Information.

### Genomic Integration

Genomic integration was performed by homologous recombination methods. First, the pET24a integration vector was constructed, the expression cassettes were integrated and the antibiotic gene with cre/lox sites was connected to the selected upstream and downstream homologous arms.^[^ ^48^ ^]^ The fragments to be integrated were amplified from the vector and purified. 1 µg DNA was transformed into the competent *B. subtilis* cells to obtain the integrated strain. To achieve continuous gene editing, resistance markers were eliminated by transforming the Cre recombinase expression plasmid with a temperature‐sensitive origin of replication. The transformed strain was coated on LB plates with 0.1 mM IPTG to induce Cre recombinase expression. Transformants were transferred to antibiotic‐free and antibiotic‐resistant plates and incubated at 5 °C for 12 h to obtain recombinant bacteria with resistance eliminated.

### *B. subtilis* Genome‐Wide CRISPRi‐Library Construction

The CRISPRi‐library strains were constructed in *B. subtilis* 1A976 background. For every individual strain in the library, a recipient strain containing high copy plasmid pUB110 which carries *poi* and a *dcas9* expression cassette integrated into the genome, was transformed with a sgRNA plasmid library. The Poi was regulated by the strong constitutive promoter P*_amyQ'_*, and the dCas9 variant derived from *Streptococcus pyogenes* was regulated by the xylose‐inducible promoter P*_xylA_*. The P*_xylA_*‐*dcas9* sequence was amplified from plasmid pJMP1^[^ ^13^ ^]^ and integrated into the amyE locus using the genome integration method described above. For the sgRNA plasmid library, 4237 open reading frame (ORF) information from *B. subtilis* was collected using SEED viewer (https://pubseed.theseed.org/ reference sequence: NC_000964.3),^[^ ^49^ ^]^ and 6000 unique sgRNAs targeting a total of 4225 ORFs (99.7%) were designed based on these sequences. The sgRNA designs were generated using the sgRNACas9 tool,^[^ ^50^ ^]^ and all sgRNAs targeted the anti‐sense strand of the ORF within the first one‐third range. These designed oligoglycosides were synthesized using high throughput DNA chips and were linked downstream of the P*_43_* promoter of the shuttle vector pAD123. The connection of the vector was completed in *E. coli*. A total of 4 × 10^7^ monoclonal clones were obtained in *E. coli*. The NGS results showed that all sgRNAs were effectively detected and had good homogeneity, with a sequence accuracy of 89.2% (Figure S1, Supporting Information). The sgRNA library plasmid used to transform the competent strain was extracted and purified from *E. coli*. By transforming the library plasmid into the *dcas9* encoding strain, a dual plasmid‐based Poi‐CRISPRi library was finally obtained. Nontargeted sgRNA expression plasmid transformed strains were used as negative controls.

### Fluorescence Activated Cell Sorting

BD FACSARia III flow cytometry was used for flow sorting. Cells used for analysis and sorting were grown to a logarithmic period and collected by centrifugation. After resuspension and 2 washes with PBS buffer, 0.3 OD_600_ cells were harvested and transferred to a flow tube for testing. The sorting process was divided into 2 rounds, where the first round of single cells with the top 5% fluorescence values were enriched and cultured, followed by secondary sorting. The top 1% of fluorescence values of single cells were collected for re‐screening validation. A 488 nm semiconductor laser excitation and FITC (530/30 nm filtered emission) channel was used to detect split‐GFP signals. Streaming data processing was performed using BD FACSDiVa.

### 96 Well Plate Screening

Flow‐sorted collected cells or clones of *B. subtilis* library were plated onto double‐resistant LB plates containing 5 µg mL^−1^ chloramphenicol and 30 µg mL^−1^ kanamycin for overnight incubation. Single colonies were first picked into 96‐well shallow plates containing 140 µL of double‐resistant LB and then placed in the Heidolph microbial screening system at 37 °C, 750 rpm for 12 h. Subsequently, 40 µL of the bacterial fluid was aspirated and transferred to 96‐well deep plates containing 600 µL of double resistant TB and incubated at 37 °C, 750 rpm for 2 h and then incubated at 33 °C until the end of fermentation. The fermentation time was 24 h for GFP and Mcherry, 36 h for β‐Gal, and 60 h for Pfa. The fluorescence‐based screening method was implemented according to the following methods. First, the 96‐well plate was centrifuged to collect the organisms after the fermentation, then the centrifuge was resuspended and washed by PBS buffer, and the suspension of PBS was finally transferred to determine the biomass and fluorescence value. The Pfa screening method was based on the DNS method. The addition of starch substrate was performed using Eppendorf epMotion 5070 high‐throughput pipetting workstations to reduce the sampling error. The original strains were selected from the glycerol‐preserved 96‐well shallow plates for sequencing and identification after high‐expression individuals were detected. All sequencing was performed using the sanger sequencing method.

### SDS‐PAGE Analysis

One‐Step PAGE Gel Fast Preparation Kit (Vazyme) was used to prepare 12% protein gel for electrophoretic analysis (which can be used to separate proteins with molecular weights of 10–180 kDa). Extraction of the wall‐breaking supernatant (soluble protein) was performed as follows: first, 5OD_600_ *B. subtilis* cells were collected and suspended in 50 mM, pH 5.0, KH_2_PO_4 –_ Na_2_HPO_4_ buffer. 40 µL of 20 mg mL^−1^ lysozyme was added and allowed to react at 37 °C for 30 min to destroy the cell wall. The samples were then subjected to ultrasonic disruption on ice at 135 W for 7 min, with 3 s on and 2 seconds off. After disruption, the supernatant obtained by centrifugation was the wall‐breaking supernatant containing soluble proteins, and the precipitate obtained by centrifugation was the insoluble part containing inclusion bodies. 5 µL and 20 µL of SDS‐PAGE Protein Sampling Buffer (5×) were added to the wall‐breaking supernatant and wall‐breaking precipitate, respectively, and then treated in a metal bath at 100 °C for 10 min. After heat treatment, the samples were centrifuged and 8 µL of the wall‐breaking supernatant mixture and 3 µL of the wall‐breaking precipitate mixture were transferred to the gel wells and then electrophoresed at 200 V for ≈30 min. The gel was stained with Thomas Brilliant Blue at the end of electrophoresis and then boiled with a destaining solution (5% ethanol and 10% glacial acetic acid) to destain the gel 3 to 4 times. The destained protein gel was then photographed using an instrument.

### Fluorescence Analysis

The detection of GFP and mCherry fluorescence intensity were both measured using the TECAN SPARK fluorescence enzyme‐linked immunosorbent assay. 200 µL of PBS suspension bacterial solution was added to a 96‐well fluorescent enzyme‐linked immunosorbent assay plate. The GFP/split GFP fluorescence intensity was excited with a 488 nm semiconductor laser and detected with a 530/30 nm filtered emission channel. mCherry fluorescence intensity was detected with a 561 nm semiconductor laser and a PE Texas Red (610/20 nm filtered) emission channel. The relative fluorescence value was calculated using the following formula, where background fluorescence FPBG was the fluorescence value obtained by detecting strains without fluorescent protein, and background absorbance ODBG was the absorbance detected by blank culture medium.

(1) $${\left(\frac{FP}{OD}\right)}_{\mathrm{corrected}}=\frac{\left(\mathrm{FP}-FP_{\mathrm{BG}}\right)}{\left(\mathrm{OD}-OD_{\mathrm{BG}}\right)}$$

### Enzyme Activity Assay

DNS (3,5‐Dinitrosalicylic Acid) method was used for the detection of *P. furiosus* hyperthermophilic amylase (Pfa).^[^ ^51^ ^]^ The Pfa enzyme activity measurement system was as follows. 1 mL bacterial fluid was first subjected to heat treatment at 90 °C for 15 min and then centrifuged to collect the supernatant. The supernatant enzyme solution was then diluted to an appropriate multiple, and then 100 µL was transferred to a plugged tube containing 1 mL of soluble starch substrate with a concentration of 10 g L^−1^ and 0.9 mL of a 50 mM pH 5.0 citric acid‐sodium citrate buffer. After incubating at 100 °C for 10 min, 3 mL of DNS solution was added to terminate the reaction. The system was boiled for 7 min for color development and diluted to 15 mL by adding deionized water after cooling. The absorbance value was measured at 540 nm. The group that added buffer instead of enzyme solution was used as a blank control. The calculation of the enzymatic activity is based on the following formula, where ΔOD_540_ represents the D‐value between absorbance value and background absorption, and *d* represents the dilution factor.

(2) $$\begin{array}{c} \mathrm{Pfa}\;\mathrm{enzyme}\;\mathrm{activity}\left(U/\mathrm{mL}\right)=5.08\times \left(\Delta OD_{540}-0.2913\right)\times d \end{array}$$

2‐Nitrophenyl‐β‐D‐galactopyranoside (oNPG) method was employed for the detection of the enzyme activity of β‐galactosidase.^[^ ^52^ ^]^ 5 OD_600_ bacterial bodies were suspended in 1 mL pH 5.0, 50 mM phosphate buffer and treated with lysozyme at 37 °C for 30 min. Then, ultrasonic wall‐breaking treatment was performed, with a wall‐breaking time of 7 min, a power of 135 W, and a 3‐s stop for 2 s. The intracellular supernatant was collected after centrifugation and diluted to an appropriate multiple. 100 µL of enzyme solution was added to a centrifuge tube containing 1.8 mL of buffer solution. After Incubating at 50 ° C until constant temperature, 100 µL of oNPG substrate (20 mM) was added into the system. After an accurate reaction for 10 min, 1 mL of Na_2_CO_3_ (1 M) was added to terminate the reaction, and the absorbance value was measured at 420 nm. The β‐Gal enzyme activity was calculated according to the following formula. The enzyme activity is the result of the calculation with the formula.

(3) $$\begin{array}{ccc} \beta -\mathrm{Gal}\;\mathrm{enzyme}\;\mathrm{activity}(\mathrm{Um}L^{-1}) & = & 0.70\\ & & \times \left(\Delta OD_{420}+0.0003\right)\times d \end{array}$$

Colloidal chitin was employed as a reaction substrate for the assay of chitinase enzyme activity. The reaction was carried out at 60 °C, pH 6.5 for 1 h, followed by the addition of DNS to terminate the reaction, and the absorbance of A_540_ was measured after boiling to develop the color. Enzyme activity is calculated as follows.

(4) $$\begin{array}{c} \mathrm{Blchi}\;\mathrm{enzyme}\;\mathrm{activity}\left(\mathrm{Um}L^{-1}\right)=0.27\times \left(\Delta OD_{540}-0.6732\right)\times d \end{array}$$

The assessment of hyperthermic amylase activity was based on the following steps. 1% starch solution was employed as the substrate. After incubating for 5 min at 70 °C and pH 6.0, terminate the reaction with DNS and boil to develop the color. A_540_ of the reaction solution was measured and the enzymatic activity was calculated using the following formula.

(5) $$\begin{array}{ccc} \mathrm{AmyS}\;\mathrm{enzyme}\;\mathrm{activity}(\mathrm{Um}L^{-1}) & = & 10.16\\ & & \times \left(\Delta OD_{540}-0.2913\right)\times d \end{array}$$

### Cell Culturing

The shake flask culture method for *B. subtilis* is as follows: first, the recombinant *B. subtilis* was cultured in 10 mL LB medium (1% (w v^−1^) tryptone, 0.5% (w v^−1^) yeast extract, 1% (w v^−1^) NaCl) at 37 °C for 12 h to prepare the seed solution, and then transferred to a 250 mL shake flask containing 50 mL TB medium (1.2% (w v^−1^) tryptone, 2.4% (w v^−1^) yeast extract, 0.5% (w v^−1^) glycerol, 1.6% (w v^−1^) K_2_HPO_4_ 3H_2_O, 0.2% (w v^−1^) KH_2_PO_4_) with a 5% (v v^−1^) inoculation amount. The high‐density fermentation base medium is composed as follows: 1.8% (w v^−1^) soybean peptone, 0.9% yeast extract (w v^−1^), 0.5% glucose (w v^−1^), 0.268% (NH_4_)_2_SO_4_ (w v^−1^), 0.1% ammonium citrate (w v^−1^), 0.452% NaH_2_PO_4_ 2H_2_O (w v^−1^), 1.46% K_2_HPO_4_ (w v^−1^), 0.1% MgSO_4_ 7H_2_O (w v^−1^), 0.2% Na_2_SO_3_ (w v^−1^), 0.04% AlCl_3_ (w v^−1^) and 0.3% (v v^−1^) metal ion liquid TES. The replenishment medium used in for high‐density fermentation process was a 5:1 ratio of glucose and organic nitrogen source, containing 4% (v v^−1^) metal ion liquid TES. After incubating at 37 °C for 2 h, the incubation temperature was switched to 33 °C until the fermentation endpoint. The culture cycle for each model protein is consistent with the method mentioned above. The concentration of antibiotics involved in cultivation was as follows: kanamycin 30 µg mL^−1^, spectinomycin 100 µg mL^−1^, tetracycline 30 µg mL^−1^, chloramphenicol 5 µg mL^−1^. Xylose and IPTG were both added when culture samples were switched to 33 °C.

The following methods are used for high‐density fermentation of *B. subtilis*. Seed culture medium was transferred to 50 mL LB culture medium at an inoculation rate of 2% (v v^−1^) from a freshly preserved glycerol tube and incubated at 37 °C for 12 h. Then, the seed solution was added to a 3L fermentor containing 900 mL of basic culture medium with a 10% (v v^−1^) inoculation amount. The fermentation parameters were set as follows: 1.5 vvm, pH 7.0, 33 °C, and 30% dissolved oxygen. 20% (v v^−1^) phosphoric acid and ammonia water were used to adjust the pH value. The feeding process was turned on after the rebound of dissolved oxygen. The feeding flow rate was controlled to ensure that the residual sugar in the system did not exceed 0.5 g L^−1^. Sampling and measuring fermentation parameters every 4 h.

### Transcriptome Analysis

Bacteria cultured to late logarithmic growth were collected, total RNA was extracted and ribosomal RNA was removed and randomly interrupted into 200 nt fragments. The first strand of cDNA was synthesized using random primers, and the second strand of cDNA was synthesized using dUTP instead of dTTP, followed by end repair, addition of base A, and addition of sequencing junctions. The second strand of cDNA was degraded using the UNG enzyme to preserve the RNA orientation information. The library preparation was completed by PCR amplification and the library was sequenced on the Illumina NovaSeq 6000 platform using NovaSeg X Series 35B Reagent Kit (300 Cycle).

Clean reads filtering was proceeded by the following standards. First, reads with ≥10% unidentified nucleotides, and> 50% bases having phred quality scores of ≤ 20, aligned to the barcode adapter were removed in order. Then, the short read alignment tool Bowtie2 (version 2.2.8)^[^ ^53^ ^]^ was used for mapping reads to the ribosome RNA (rRNA) database. The rRNA‐mapped reads then will be removed. The retained reads were aligned with the reference genome using Bowtie2 (version 2.2.8) to identify known genes. Gene expression was calculated using RSEM.^[^ ^54^ ^]^

The gene expression level was further normalized to eliminate the influence of different gene lengths and amounts of sequencing data on the calculation of gene expression using the fragments per kilobase of transcript per million (FPKM) mapped reads method. The edgeR package (http://www.r‐project.org/) was used to identify differentially expressed genes (DEGs) across samples with fold changes ≥ 2 and a false discovery rate‐adjusted *p* (*q* value) < 0.05. The *p*‐value for this hypothesis test was calculated as:

(6) $$P\;=\;1-\sum_{i\;=\;0}^{m-1}\frac{\left(\begin{array}{c} M\\ i \end{array}\right)\left(\begin{array}{c} N-M\\ n-i \end{array}\right)}{\left(\begin{array}{c} N\\ n \end{array}\right)}$$

where *N* is the number of transcripts with GO annotations in all Unigenes; *n* is the number of differentially expressed transcripts in *N*; *M* is the number of transcripts annotated with a particular GO term in all Unigenes, and *m* is the number of differentially expressed transcripts annotated with a particular GO term. The calculated *p*‐value was corrected by FDR to obtain the Q‐value.

When performing GO analysis, differentially expressed transcripts were mapped to each term of the GO database (http://www.geneontology.org/). Then the number of transcripts in each term was counted to obtain the list of transcripts with a certain GO function and the statistics of the number of transcripts. Hypergeometric tests were then applied to identify GO entries that were significantly enriched in differentially expressed transcripts compared to the whole transcriptome background. With Q‐value ≤ 0.05 as the threshold, a GO term satisfying this condition was defined as a GO term that was significantly enriched in differentially expressed transcripts.

### Statistical Analysis

All experiments were repeated 3 times. Values and error bars reflect the mean ± s.d. of 3 independent biological replicates (*n* = 3). Differences between multiple sets of data were compared using one‐way ANOVA followed by Tukey's test. ^*^ *p *< 0.05, ^**^ *p *< 0.01, and ^***^ *p *< 0.001.

## Conflict of Interest

The authors declare no conflict of interest.

## Supporting information

Supporting Information

## Data Availability

The data that support the findings of this study are available from the corresponding author upon reasonable request.
