# Supplementary material for: Genome‐Wide CRISPRi Screening of Key Genes for Recombinant Protein Expression in Bacillus Subtilis
Source: Adv Sci (Weinh). 2024 Jul 1;11(33):2404313. doi: 10.1002/advs.202404313 (PMC11434012; doi:10.1002/advs.202404313)
Supplement: Supplementary file 1 — Supporting Information [file ADVS-11-2404313-s001.docx]

Supporting Information

**Genome-wide CRISPRi screening of key genes for recombinant protein expression in *Bacillus subtilis***

*Xuyang Zhu, Hui Luo, Xinrui Yu, Huihui Lv, Lingqia Su, Kang Zhang* and Jing Wu**

**Supplementary Figures**


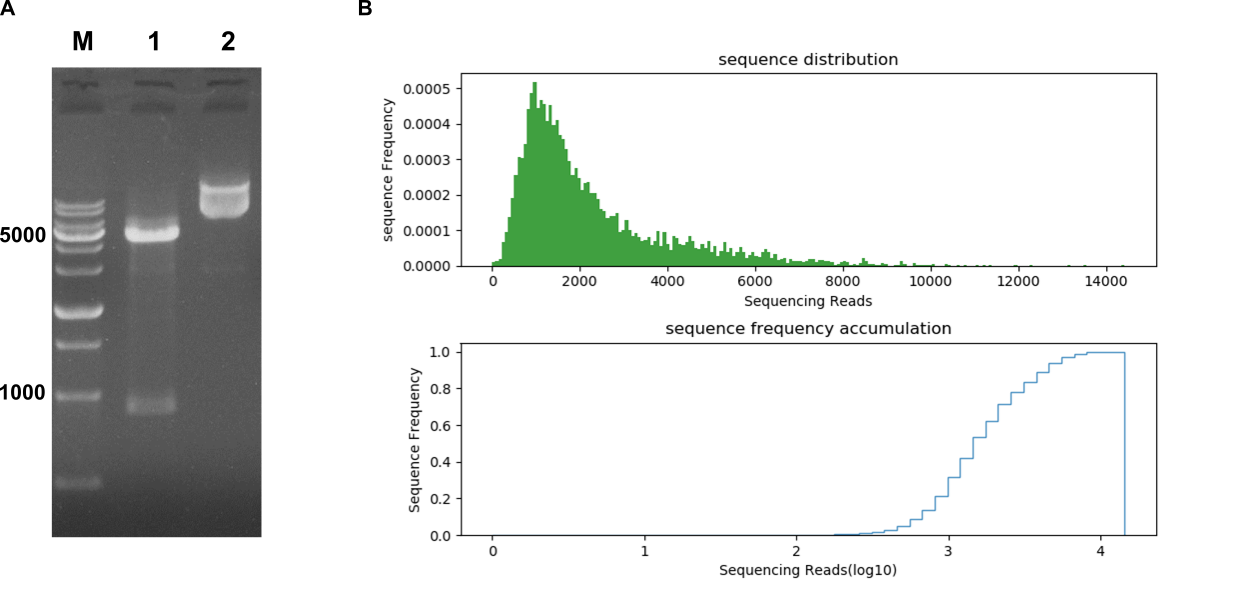


**Supplementary Figure S1.** CRISPRi sgRNA library quality analysis. A, Plasmid digestion analysis of libraries. Lane M: DNA Marker; Lane 1: Plasmid digested by KpnI and BglI; Lane 2: Plasmid DNA; Lane 2: Plasmid DNA. B, Analysis of library homogeneity data. The upper graph shows that the sequencing reads of the sgRNAs satisfy a normal distribution, which in turn has a high degree of homogeneity and a low degree of dispersion.

The lower graph shows the cumulative distribution of the frequency of Reads counts for different sgRNAs, which also shows the better homogeneity of the library.


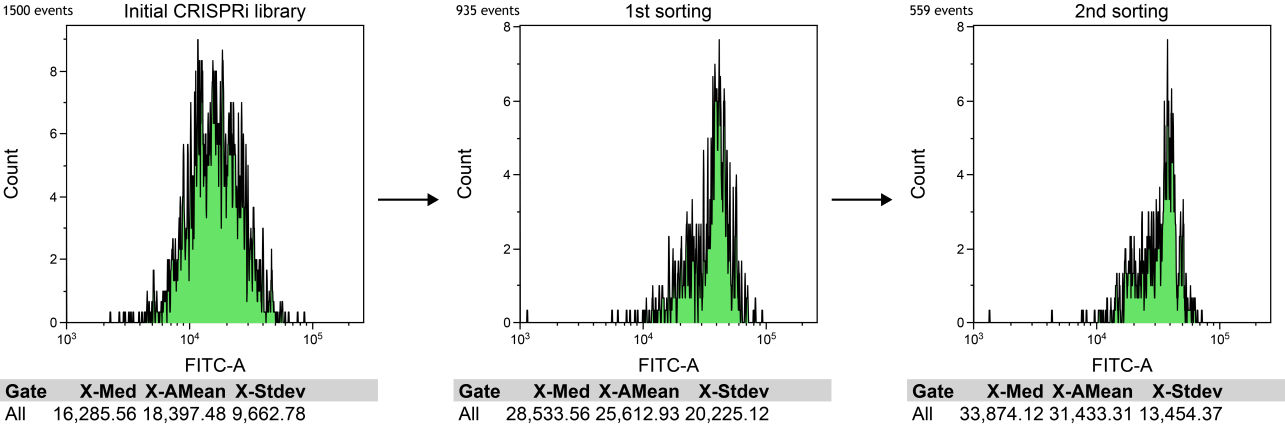


**Supplementary Figure S2.** Split-GFP based β-Gal flow-through sorting with multiple rounds of enrichment. Two rounds of enrichment sorting were performed on the initial CRISPRi library, taking the top 5 % and 1 % of fluorescence intensity, respectively, and the enriched cells were subjected to a reflux assay to determine the fluorescence of the cell populations; X-Med represents the median fluorescence value; X-Amean represents the mean fluorescence value; and X-Stdev represents the standard deviation.


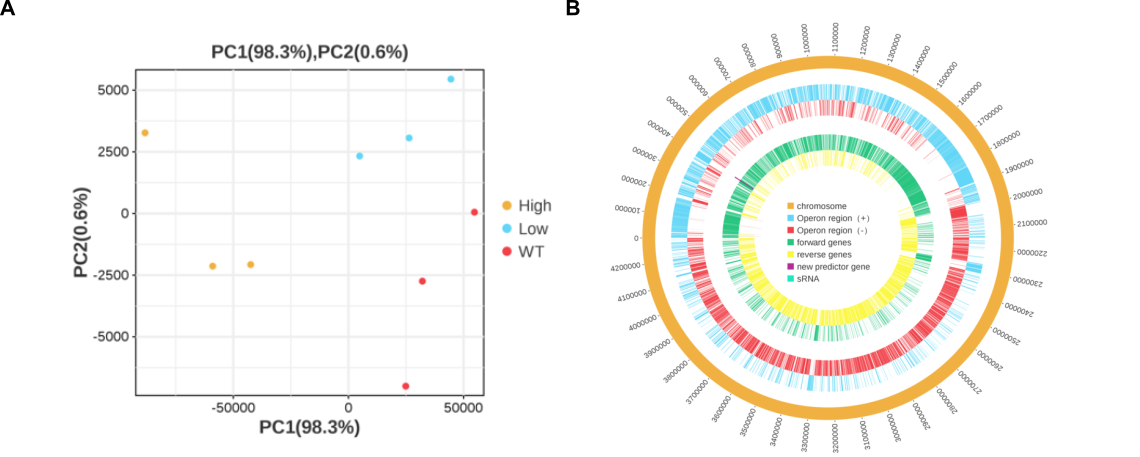


**Supplementary Figure S3.** Transcript sample relationships and gene structure. A, Principal component analysis (PCA, Principal Component Analysis) was carried out based on gene expression information, three groups of transcript samples show their respective regions of aggregation. B, Chromosome gene structure. The outermost circle (orange) represents the chromosome, with a scale unit of 1K bp. The next inner circle (blue for the positive strand, red for the negative strand) represents the manipulator region. The innermost circle is for individual genes, with green and yellow representing known genes; purple represents newly predicted genes, where sRNAs are indicated in aquamarine.


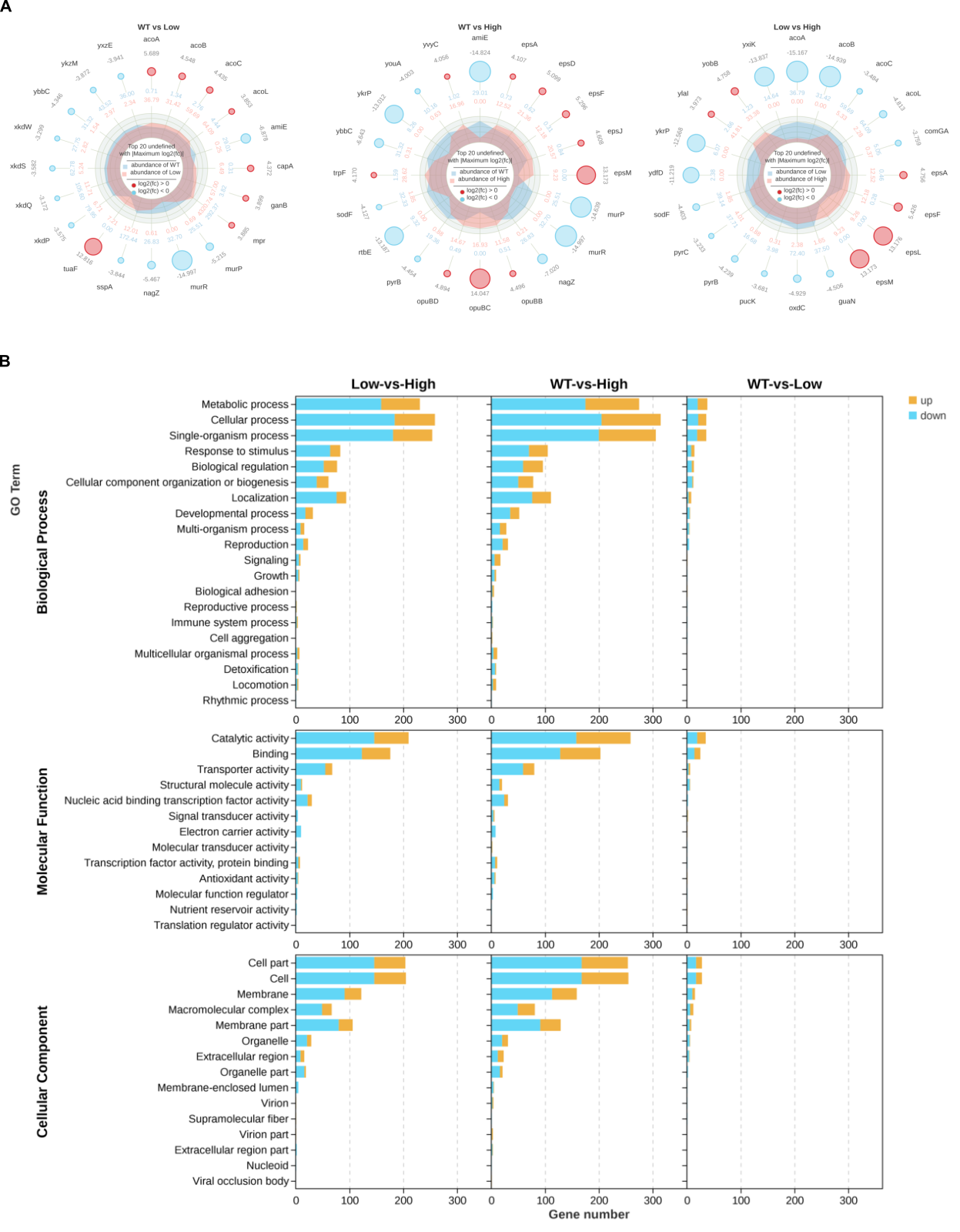


**Supplementary Figure S4.** Differential analysis of WS9MLHS transcripts. A, Information on the TOP 20 genes with the highest degree of variation. The default screening conditions for significantly different genes were |log_2_FC| ≥ 1 and FDR < 0.05, Outermost circle: numbers represent log_2_(FC) values, i.e. log with 2 as the logarithm of the bottom difference multiple; red circle and sky blue circle: up-regulated genes and down-regulated genes, the size of the circle varies according to the size of the log_2_(FC) value; third circle: data in the outer circle represent the average expression of sample A; data in the inner circle represent the average expression of sample B; irregular shapes in the circle: the abundance of expression of samples A and B on each axis.B, Differential GO enrichment analysis.

**Supplementary Table S1. Strains and plasmids used in this study**

| Strains and plasmids | Genotype description | Source |
| --- | --- | --- |
| *E. coli* JM109 | *thi, recA1, gyrA96, endA1, supE44* | Takara |
| *B. subtilis* 1A976 | *his, nprE18, aprE3, eglSΔ102, bglT/bglSΔEV, lacA*::P*_xylA_*-*comK* | Laboratory |
| *B. subtilis* 1A976D | Constructed by WS9C, *amyE*::P*_xylA_*-*dcas9* | This study |
| *B. subtilis* 1A976G | Constructed by WS9D, *epr*::P*_amyQ’_*-*gfpmut3* | This study |
| *Bacillus subtilis* WS9C | Derived from an undomesticated strain, *ΔsrfC, ΔspoIIAC, ΔnprE, ΔamyE, ΔaprE, ΔnprB, Δbpr, Δmpr, Δepr, amyE*::P*_xylA_*-*comK* | Laboratory |
| *Bacillus subtilis* WS9D | Constructed by WS9C, *epr*::P*_grac100_*-*dcas9-ω* | This study |
| *Bacillus subtilis* WS9FP | Constructed by WS9D, nprE::P*_spoVG_*-*mcherry*-P*_amyQ’_*-*gfpmut3* | This study |
| *Bacillus subtilis* WS9NT | Constructed by WS9D, *lacA*::P*_43_*-*sgRNA-NT* | This study |
| *Bacillus subtilis* WS9MLHS | Constructed by WS9D, *lacA*::P*_43_*-*sgRNA-murR-lplC, mpr*::P*_43_*-*sgRNA-hrcA-S2* | This study |
| pUB110 | Kana^r^, *B. subtilis* expression plasmid, containing pUBori | Takara |
| pAD123 | Amp^r^, Cm^r^, *B. subtilis-E. coli* shuttle plasmid, containing Rep pTA1060 and pBR322ori | BSGC |
| pJMP1 | Amp^r^, containing Spdcas9 under P*_xylA_* promoter | BSGC |
| pET24a | Kan^r^, *E. coli* expression vector | Takara |
| pUB110-pfa | Kana^r^, containing pfa under P*_amyQ’_* promoter | This study |
| pUB110-gal-split-gfp | Kanar, containing *β-gal-gfp_11_-gfp_1-10_* under P*_amyQ’_* promoter | This study |
| pAD123-sgNT | Amp^r^, Cm^r^, *B. subtilis-E. coli* shuttle plasmid, containing non-targeting sgRNA | This study |
| pAD123-sgG1 | Amp^r^, Cm^r^, *B. subtilis-E. coli* shuttle plasmid, containing sgRNA targeting gfp at 40nt | This study |
| pAD123-sgG2 | Amp^r^, Cm^r^, *B. subtilis-E. coli* shuttle plasmid, containing sgRNA targeting gfp at 169nt | This study |
| pAD123-sgG3 | Amp^r^, Cm^r^, *B. subtilis-E. coli* shuttle plasmid, containing sgRNA targeting gfp at 172nt | This study |
| pAD123-sgG4 | Amp^r^, Cm^r^, *B. subtilis-E. coli* shuttle plasmid, containing sgRNA targeting gfp at 263nt | This study |
| pAD123-sgcgeA | Amp^r^, Cm^r^, *B. subtilis-E. coli* shuttle plasmid, containing sgRNA targeting *cgeA* | This study |
| pAD123-sgyisP | Amp^r^, Cm^r^, *B. subtilis-E. coli* shuttle plasmid, containing sgRNA targeting *yisP* | This study |
| pAD123-sgfliM | Amp^r^, Cm^r^, *B. subtilis-E. coli* shuttle plasmid, containing sgRNA targeting *fliM* | This study |
| pAD123-sgpxpI | Amp^r^, Cm^r^, *B. subtilis-E. coli* shuttle plasmid, containing sgRNA targeting *pxpI* | This study |
| pAD123-sgmurR | Amp^r^, Cm^r^, *B. subtilis-E. coli* shuttle plasmid, containing sgRNA targeting *murR* | This study |
| pAD123-sglplC | Amp^r^, Cm^r^, *B. subtilis-E. coli* shuttle plasmid, containing sgRNA targeting *lplC* | This study |
| pAD123-sggcvPA | Amp^r^, Cm^r^, *B. subtilis-E. coli* shuttle plasmid, containing sgRNA targeting *gcvPA* | This study |
| pAD123-sghrcA | Amp^r^, Cm^r^, *B. subtilis-E. coli* shuttle plasmid, containing sgRNA targeting *hrcA* | This study |
| pAD123-sgyozI | Amp^r^, Cm^r^, *B. subtilis-E. coli* shuttle plasmid, containing sgRNA targeting *yozI* | This study |
| pAD123-sgyoaQ | Amp^r^, Cm^r^, *B. subtilis-E. coli* shuttle plasmid, containing sgRNA targeting *yoaQ* | This study |
| pAD123-sgyndF | Amp^r^, Cm^r^, *B. subtilis-E. coli* shuttle plasmid, containing sgRNA targeting *yndF* | This study |
| pAD123-sgybaC | Amp^r^, Cm^r^, *B. subtilis-E. coli* shuttle plasmid, containing sgRNA targeting *ybaC* | This study |
| pET-dcas9 | Kan^r^, containing dcas9 under P*_xylA_* promoter and *amyE* homology arms. | This study |
| pET-dcas9-ω | Kan^r^, containing dcas9-ω under P*_grac100_* promoter and *epr* homology arms. | This study |
| pET-sgNT | Kan^r^, containing non-targeting sgRNA under P*_43_* promoter and *lacA* homology arms. | This study |
| pET-sgmurR | Kan^r^, containing sgRNA targeting *murR* under P*_43_* promoter and *lacA* homology arms. | This study |
| pET-sglplC | Kan^r^, containing sgRNA targeting *lplC* under P*_43_* promoter and *lacA* homology arms. | This study |
| pET-sggcvPA | Kan^r^, containing sgRNA targeting *gcvPA* under P*_43_* promoter and *lacA* homology arms. | This study |
| pET-sghrcA | Kan^r^, containing sgRNA targeting *hrcA* under P*_43_* promoter and *lacA* homology arms. | This study |
| pET-sgyozI | Kan^r^, containing sgRNA targeting *yozI* under P*_43_* promoter and *lacA* homology arms. | This study |
| pET-sgyoaQ | Kan^r^, containing sgRNA targeting *yoaQ* under P*_43_* promoter and *lacA* homology arms. | This study |
| pET-sgyndF | Kan^r^, containing sgRNA targeting *yndF* under P*_43_* promoter and *lacA* homology arms. | This study |
| pET-sgybaC | Kan^r^, containing sgRNA targeting *ybaC* under P*_43_* promoter and *lacA* homology arms. | This study |
| pET-sgmurR-lplC | Kan^r^, containing sgRNA targeting *murR* and *lplC* under P*_43_* promoter and *lacA* homology arms. | This study |
| pET-sgmurR-hrcA | Kan^r^, containing sgRNA targeting *murR* and *hrcA* under P*_43_* promoter and *lacA* homology arms. | This study |
| pET-sgmurR-yozI | Kan^r^, containing sgRNA targeting *murR* and *yozI* under P*_43_* promoter and *lacA* homology arms. | This study |
| pET-sgmurR-yoaQ | Kan^r^, containing sgRNA targeting *murR* and *yoaQ* under P*_43_* promoter and *lacA* homology arms. | This study |
| pET-sghrcA2 | Kan^r^, containing sgRNA targeting *hrcA* under P*_43_* promoter and *mpr* homology arms. | This study |
| pET-sgM1 | Kan^r^, containing sgRNA targeting *mcherry* at 48nt from TSS under P*_43_* promoter and *lacA* homology arms. | This study |
| pET-sgM2 | Kan^r^, containing sgRNA targeting *mcherry* at 68nt from TSS under P*_43_* promoter and *lacA* homology arms. | This study |
| pET-sgM3 | Kan^r^, containing sgRNA targeting *mcherry* at 69nt from TSS under P*_43_* promoter and *lacA* homology arms. | This study |
| pET-sgM4 | Kan^r^, containing sgRNA targeting *mcherry* at 76nt from TSS under P*_43_* promoter and *lacA* homology arms. | This study |
| pET-sgM5 | Kan^r^, containing sgRNA targeting *mcherry* at 97nt from TSS under P*_43_* promoter and *lacA* homology arms. | This study |
| pET-sgM6 | Kan^r^, containing sgRNA targeting *mcherry* at 201nt from TSS under P*_43_* promoter and *lacA* homology arms. | This study |
| pET-sghrcA-S1 | Kan^r^, containing sgRNA targeting *hrcA* and *pfa* at 95nt from TSS under P*_43_* promoter and *mpr* homology arms. | This study |
| pET-sghrcA-S2 | Kan^r^, containing sgRNA targeting *hrcA* and *pfa* at 110nt from TSS under P*_43_* promoter and *mpr* homology arms. | This study |
| pET-sghrcA-S3 | Kan^r^, containing sgRNA targeting *hrcA* and *pfa* at 129nt from TSS under P*_43_* promoter and *mpr* homology arms. | This study |
| pET-sghrcA-A1 | Kan^r^, containing sgRNA targeting *hrcA* and *pfa* at 135nt from TSS under P*_43_* promoter and *mpr* homology arms. | This study |

**Supplementary Table S2. The primers used in this study**

| Primer | Sequence（5’-3’） |
| --- | --- |
| Fdcas | AAGGAGGAAGGATCCATGGATAAGAAATACTCAATAGGCTTAGC |
| Rdcas | TCTAGAAGCTGCAGAACCGTCACCTCCTAGCTGACTCAAATC |
| FyloH(ω) | GGTTCTGCAGCTTCTAGAATGTTAGATCCGTCAATTGATTCTTTAATG |
| RyloH(ω) | AGATCCTTACTCGAGCTATTCGCGGTCTTCCTTTTC |
| Fpfa | AAAAATCAAATAAGGAGTGTCAAGAATGAAACTGGCAATCACAGCG |
| Rpfa | AGCTTGGAGGTGTTTTTTTATTACCTTACCCTACCCCGCAATATGAC |
| Fgal | AAAAATCAAATAAGGAGTGTCAAGAATGCGTCGTATTAACTTTAACG |
| Rgal | GAGATCCTCCGCCAGAGCCTCCGCCCTGATCAGCAGAGGCCGTC |
| Fgfp11 | CTTTACGACGGCCTCTGCTGATCAGGGCGGAGGCTCTGGCG |
| Rgfp11 | TCTTGACACTCCTTTCATGTGATGCCTGCTGCATTAAC |
| Fgfp1-10 | GCATCACATGAAAGGAGTGTCAAGAATGTCTAAAGGCGAAGAACTGTTTAC |
| Rgfp1-10 | TTGGAGGTGTTTTTTTATTACCTTATTTTTCATTCGGATCTTTAGACAGAAC |
| FsgNT | TTATGTAAAATATAAGTTTTAGAGCTAGAAATAGCAAGTTAAAATAAGG |
| RsgNT | TTCTAGCTCTAAAACTTATATTTTACATAATCGCGCGC |
| FsgG1 | CCATCTAATTCAACAAGAATGTTTTAGAGCTAGAAATAGCAAGTTAAAATAAGG |
| RsgG1 | ATTCTTGTTGAATTAGATGGTTATATTTTACATAATCGCGCGC |
| FsgG2 | GTAGTGACAAGTGTTGGCCAGTTTTAGAGCTAGAAATAGCAAGTTAAAATAAGG |
| RsgG2 | TGGCCAACACTTGTCACTACTTATATTTTACATAATCGCGCGC |
| FsgG3 | CCAACACTTGTCACTACTTTGTTTTAGAGCTAGAAATAGCAAGTTAAAATAAGG |
| RsgG3 | AAAGTAGTGACAAGTGTTGGTTATATTTTACATAATCGCGCGC |
| FsgG4 | CTGTACATAACCTTCGGGCAGTTTTAGAGCTAGAAATAGCAAGTTAAAATAAGG |
| RsgG4 | TGCCCGAAGGTTATGTACAGTTATATTTTACATAATCGCGCGC |
| FsgcgeA | TCTGTTGCAGTTGGAAATAAGTTTTAGAGCTAGAAATAGCAAGTTAAAATAAGG |
| RsgcgeA | TTATTTCCAACTGCAACAGATTATATTTTACATAATCGCGCGC |
| FsgyisP | GATAATGCTTTGAAACAGGCGTTTTAGAGCTAGAAATAGCAAGTTAAAATAAGG |
| RsgyisP | GCCTGTTTCAAAGCATTATCTTATATTTTACATAATCGCGCGC |
| FsgfliM | GCAGTGCATCTATTTCATTTGTTTTAGAGCTAGAAATAGCAAGTTAAAATAAGG |
| RsgfliM | AAATGAAATAGATGCACTGCTTATATTTTACATAATCGCGCGC |
| FsgpxpI | GTAGCCGCCGGACATGCCTGGTTTTAGAGCTAGAAATAGCAAGTTAAAATAAGG |
| RsgpxpI | CAGGCATGTCCGGCGGCTACTTATATTTTACATAATCGCGCGC |
| FsgmurR | GTGCTTTCGATTGCTTTGTGGTTTTAGAGCTAGAAATAGCAAGTTAAAATAAGG |
| RsgmurR | CACAAAGCAATCGAAAGCACTTATATTTTACATAATCGCGCGC |
| FsglplC | GAGTTCCGCTTCTGTCGCAAGTTTTAGAGCTAGAAATAGCAAGTTAAAATAAGG |
| RsglplC | TTGCGACAGAAGCGGAACTCTTATATTTTACATAATCGCGCGC |
| FsghrcA | TATTAAATCGGCACAGCCGGGTTTTAGAGCTAGAAATAGCAAGTTAAAATAAGG |
| RsghrcA | CCGGCTGTGCCGATTTAATATTATATTTTACATAATCGCGCGC |
| FsgyozI | TACTCACCATCATTGTTTAAGTTTTAGAGCTAGAAATAGCAAGTTAAAATAAGG |
| RsgyozI | TTAAACAATGATGGTGAGTATTATATTTTACATAATCGCGCGC |
| FsggcvPA | CCACAATGACAGGCTGATAGGTTTTAGAGCTAGAAATAGCAAGTTAAAATAAGG |
| RsggcvPA | CTATCAGCCTGTCATTGTGGTTATATTTTACATAATCGCGCGC |
| FsgyoaQ | TGATACTCGCCTTTATACAAGTTTTAGAGCTAGAAATAGCAAGTTAAAATAAGG |
| RsgyoaQ | TTGTATAAAGGCGAGTATCATTATATTTTACATAATCGCGCGC |
| FsgyndF | AGACATACTATGACCATAGCGTTTTAGAGCTAGAAATAGCAAGTTAAAATAAGG |
| RsgyndF | GCTATGGTCATAGTATGTCTTTATATTTTACATAATCGCGCGC |
| FsgybaC | TGTCTAACATATCCGATTTGGTTTTAGAGCTAGAAATAGCAAGTTAAAATAAGG |
| RsgybaC | CAAATCGGATATGTTAGACATTATATTTTACATAATCGCGCGC |
| FM1 | TAGGATATAGTTACACAATTGTTTTAGAGCTAGAAATAGCAAGTTAAAATAAGG |
| RM1 | AATTGTGTAACTATATCCTATTATATTTTACATAATCGCGCGC |
| FM2 | AGGTCATAAAGCTTTCATTTGTTTTAGAGCTAGAAATAGCAAGTTAAAATAAGG |
| RM2 | AAATGAAAGCTTTATGACCTTTATATTTTACATAATCGCGCGC |
| FM3 | GGTCATAAAGCTTTCATTTCGTTTTAGAGCTAGAAATAGCAAGTTAAAATAAGG |
| RM3 | GAAATGAAAGCTTTATGACCTTATATTTTACATAATCGCGCGC |
| FM4 | AAGCTTTCATTTCGGGCATAGTTTTAGAGCTAGAAATAGCAAGTTAAAATAAGG |
| RM4 | TATGCCCGAAATGAAAGCTTTTATATTTTACATAATCGCGCGC |
| FM5 | GGTAAATAAAAAAGTGATCCGTTTTAGAGCTAGAAATAGCAAGTTAAAATAAGG |
| RM5 | GGATCACTTTTTTATTTACCTTATATTTTACATAATCGCGCGC |
| FM6 | ACTTCAACACAAGATCTCGCGTTTTAGAGCTAGAAATAGCAAGTTAAAATAAGG |
| RM6 | GCGAGATCTTGTGTTGAAGTTTATATTTTACATAATCGCGCGC |
| FS1 | GCGGCGTTCTGTTTCTGCTTGTTTTAGAGCTAGAAATAGCAAGTTAAAATAAGG |
| RS1 | AAGCAGAAACAGAACGCCGCTTATATTTTACATAATCGCGCGC |
| FS2 | TCGGTATGTGATTGTGAAGCGTTTTAGAGCTAGAAATAGCAAGTTAAAATAAGG |
| RS2 | GCTTCACAATCACATACCGATTATATTTTACATAATCGCGCGC |
| FS3 | GAAGCTGGCTTACAGAAGAGGTTTTAGAGCTAGAAATAGCAAGTTAAAATAAGG |
| RS3 | CTCTTCTGTAAGCCAGCTTCTTATATTTTACATAATCGCGCGC |
| FA1 | AAACAGAACGCCGCCGATCCGTTTTAGAGCTAGAAATAGCAAGTTAAAATAAGG |
| RA1 | GGATCGGCGGCGTTCTGTTTTTATATTTTACATAATCGCGCGC |

*The underlined portions are homology arms.

**Plasmids for recombinant protein expression**

**Pfa expression plasmid (pUB110-pfa) sequence**

The PamyQ' promoter is coloured purple and pfa is lighted blue.

GGATCGGCGGCGTTCTGTTTCTGCTTCGGTATGTGATTGTGAAGCTGGCTTACAGAAGAGCGGTAAAAGAAGAAATAAAAAAGAAATCATCTTTTTTGTTTGGAAAGCGAGGGAAGCGTTCACAGTTTCGGGCAGCTTTTTTTATAGGAACATTGATTTGTATTCACTCTGCCAAGTTGTTTTGATAGAGTGATTGTGATAATTTTAATGTAAGCGATAACAAAATTCTCCAGTCTTCACATCGGTTTGAAAGGAGGAAGCGGAAGAATGAAGTAAGAGGGATTTTTGACTCCGAAGTAAGTCTTCAAAAAATCAAATAAGGAGTGTCAAGAATGAAACTGGCAATCACAGCGAAAGCGGCGGCCGGTGCACATATGGCCAAATACTTAGAACTCGAAGAGGGCGGTGTTATAATGCAAGCATTCTATTGGGATGTTCCAGGAGGCGGAATCTGGTGGGACCACATCAGAAGCAAGATTCCTGAATGGTACGAGGCCGGCATAAGCGCCATCTGGCTGCCACCTCCTTCTAAAGGAATGAGTGGAGGATATAGTATGGGGTACGACCCATACGACTACTTTGACCTGGGTGAATATTATCAGAAAGGCACAGTAGAGACACGCTTTGGATCAAAGGAGGAATTAGTTCGGTTGATTCAAACAGCTCATGCTTATGGGATTAAGGTTATCGCCGATGTGGTTATCAACCATAGAGCTGGAGGCGATCTTGAATGGAACCCATTCGTTGGTGATTATACATGGACAGATTTTTCTAAGGTTGCTTCTGGAAAGTATACTGCGAACTACTTGGACTTCCACCCAAATGAGCTTCACTGTTGTGACGAGGGTACCTTTGGTGGGTTCCCGGATATATGTCACCACAAGGAGTGGGATCAATACTGGCTTTGGAAATCAAACGAATCTTACGCGGCATATTTACGGAGCATAGGGTTCGACGGGTGGCGTTTCGACTACGTGAAGGGATACGGTGCTTGGGTGGTTCGCGACTGGCTGAACTGGTGGGGAGGTTGGGCAGTCGGAGAGTATTGGGACACGAACGTTGACGCTTTGCTTTCCTGGGCTTACGAGAGCGGTGCAAAGGTCTTTGACTTTCCACTTTATTATAAGATGGACGAGGCTTTTGATAACAATAATATTCCTGCATTAGTATACGCGTTACAAAACGGTCAGACGGTTGTAAGCAGAGATCCATTCAAGGCAGTTACATTTGTCGCCAACCATGATACGGACATCATTTGGAACAAGTACCCTGCCTACGCATTTATCTTAACTTACGAGGGTCAACCAGTAATTTTCTATAGAGATTTTGAAGAATGGTTGAACAAGGATAAACTTATTAACCTCATCTGGATACACGACCACCTGGCTGGTGGGTCCACGACAATAGTCTACTACGACAACGATGAGTTAATCTTTGTTCGGAACGGAGATTCCCGCCGCCCAGGTTTAATCACTTACATAAATCTGTCCCCTAACTGGGTGGGGCGTTGGGTGTATGTCCCGAAATTTGCAGGTGCCTGTATACATGAGTATACTGGCAATCTTGGAGGCTGGGTAGATAAACGTGTTGACAGTAGTGGATGGGTTTACTTGGAGGCTCCGCCGCATGATCCTGCCAACGGGTACTATGGATATTCCGTGTGGTCATATTGCGGGGTAGGGTAAGGTAATAAAAAAACACCTCCAAGCTGAGTGCGGGTATCAGCTTGGAGGTGCGTTTATTTTTTCAGCCGTATGACAAGGTCGGCATCAGGTGTGACAAATACGGTATGCTGGCTGTCATAGGTGACAAATCCGGGTTTTGCGCCGTTTGGCTTTTTCACATGTCTGATTTTTGTATAATCAACAGGCACGGAGCCGGAATCTTTCGCCTTGGAAAAATAAGCGGCGATCGTAGCTGCTTCCAATATGGATTGTTCATCGGGATCGCTGCTTTTAATCACAACGTGGGAGATCCAGTTGCTCAAAAAAATCTCGGTCAGATGTTACTAGCAACTCATTTACAAGAACAGCATCTTTCCTCGTTTTTCTTGTACCTGTTTTTTGTGATTCAATAATTTCTTTGACACGTTCGTTGTAATCAATATTTTTATCATTTTTCAAATCATAATTTTCACGTGTTCGCTCATGGTCAATATCATCATTCGTTCTACTTTTTCGCTCTCTTTGATTATGAAATTGCATGCCTTTTAGTCCAGCTGATTTCACTTTTTGCATTCTACAAACTGCATAACTCATATGTAAATCGCTCCTTTTTAGGTGGCACAAATGTGAGGCATTTTCGCTCTTTCCGGCAACCACTTCCAAGTAAAGTATAACACACTATACTTTATATTCATAAAGTGTGTGCTCTGCGAGGCGCGGCAGTGCCGACCAAAACCATAAAACCTTTAAGACCTTTCTTTTTTTTACGAGAAAAAAGAAACAAAAAAACCTGCCCTCTGCCACCTCAGCAAAGGGGGGTTTTGCTCTCGTGCTCGTTTAAAAATCAGCAAGGGACAGGTAGTATTTTTTGAGAAGATCACTCAAAAAATCTCCACCTTTAAACCCTTGCCAATTTTTATTTTGTCCGTTTTGTCTAGCTTACCGAAAGCCAGACTCAGCAAGAATAAAATTTTTATTGTCTTTCGGTTTTCTAGTGTAACGGACAAAACCACTCAAAATAAAAAAGATACAAGAGAGGTCTCTCGTATCTTTTATTCAGCAATCGCGCCCGATTGCTGAACAGATTAATAATAGATTTTAGCTTTTTATTTGTTGAAAAAAGCTAATCAAATTGTTGTCGGGATCAATTACTGCAAAGTCTCGTTCATCCCACCACTGATCTTTTAATGATGTATTGGGGTGCAAAATGCCCAAAGGCTTAATATGTTGATATAATTCATCAATTCCCTCTACTTCAATGCGGCAACTAGCAGTACCAGCAATAAACGACTCCGCACCTGTACAAACCGGTGAATCATTACTACGAGGCGCCAGCCTTCATCACTTGCCTCCCATAGATGAATCCGAACCTCATTACACATTAGAACTGCGAATCCATCTTCATGGTGAACCAAAGTGAAACCTACTTTATCCCAATAAAAACCTATACTCTTTTTAATATCCCCGACTGGCAATGCCGGGATAGACTGTAACATTCTCACGCATAAAATCCCCTTTCATTTTCTAATGTAAATCTATTACCTTATTATTAATTCAATTCGCTCATAATTAATCCTTTTTCTTATTACGCAAAATGGCCCGATTTAAGCACACCCTTTATTCCGTTAATGCGCCATGACAGCCATGATAATTACTAATACTAGGAGAAGTTAATAAATACGTAACCAACATGATTAACAATTATTAGAGGTCATCGTTCAAAATGGTATGCGTTTTGACACATCCACTATATATCCGTGTCGTTCTGTCCACTCCTGAATCCCATTCCAGAAATTCTCTAGCGATTCCAGAAGTTTCTCAGAGTCGGAAAGTTGACCAGACATTACGAACTGGCACAGATGGTCATAACCTGAAGGAAGATCTGATTGCTTAACTGCTTCAGTTAAGACCGAAGCGCTCGTCGTATAACAGATGCGATGATGCAGACCAATCAACATGGCACCTGCCATTGCTACCTGTACAGTCAAGGATGGTAGAAATGTTGTCGGTCCTTGCACACGAATATTACGCCATTTGCCTGCATATTCAAACAGCTCTTCTACGATAAGGGCACAAATCGCATCGTGGAACGTTTGGGCTTCTACCGATTTAGCAGTTTGATACACTTTCTCTAAGTATCCACCTGAATCATAAATCGGCAAAATAGAGAAAAATTGACCATGTGTAAGCGGCCAATCTGATTCCACCTGAGATGCATAATCTAGTAGAATCTCTTCGCTATCAAAATTCACTTCCACCTTCCACTCACCGGTTGTCCATTCATGGCTGAACTCTGCTTCCTCTGTTGACATGACACACATCATCTCAATATCCGAATAGGGCCCATCAGTCTGACGACCAAGAGAGCCATAAACACCAATAGCCTTAACATCATCCCCATATTTATCCAATATTCGTTCCTTAATTTCATGAACAATCTTCATTCTTTCTTCTCTAGTCATTATTATTGGTCCATTCACTATTCTCATTCCCTTTTCAGATAATTTTAGATTTGCTTTTCTAAATAAGAATATTTGGAGAGCACCGTTCTTATTCAGCTATTAATAACTCGTCTTCCTAAGCATCCTTCAATCCTTTTAATAACAATTATAGCATCTAATCTTCAACAAACTGGCCCGTTTGTTGAACTACTCTTTAATAAAATAATTTTTCCGTTCCCAATTCCACATTGCAATAATAGAAAATCCATCTTCATCGGCTTTTTCGTCATCATCTGTATGAATCAAATCGCCTTCTTCTGTGTCATCAAGGTTTAATTTTTTATGTATTTCTTTTAACAAACCACCATAGGAGATTAACCTTTTACGGTGTAAACCTTCCTCCAAATCAGACAAACGTTTCAAATTCTTTTCTTCATCATCGGTCATAAAATCCGTATCCTTTACAGGATATTTTGCAGTTTCGTCAATTGCCGATTGTATATCCGATTTATATTTATTTTTCGGTCGAATCATTTGAACTTTTACATTTGGATCATAGTCTAATTTCATTGCCTTTTTCCAAAATTGAATCCATTGTTTTTGATTCACGTAGTTTTCTGTATTCTTAAAATAAGTTGGTTCCACACATACCAATACATGCATGTGCTGATTATAAGAATTATCTTTATTATTTATTGTCACTTCCGTTGCACGCATAAAACCAACAAGATTTTTATTAATTTTTTTATATTGCATCATTCGGCGAAATCCTTGAGCCATATCTGACAAACTCTTATTTAATTCTTCGCCATCATAAACATTTTTAACTGTTAATGTGAGAAACAACCAACGAACTGTTGGCTTTTGTTTAATAACTTCAGCAACAACCTTTTGTGACTGAATGCCATGTTTCATTGCTCTCCTCCAGTTGCACATTGGACAAAGCCTGGATTTACAAAACCACACTCGATACAACTTTCTTTCGCCTGTTTCACGATTTTGTTTATACTCTAATATTTCAGCACAATCTTTTACTCTTTCAGCCTTTTTAAATTCAAGAATATGCAGAAGTTCAAAGTAATCAACATTAGCGATTTTCTTTTCTCTCCATGGTCTCACTTTTCCACTTTTTGTCTTGTCCACTAAAACCCTTGATTTTTCATCTGAATAAATGCTACTATTAGGACACATAATATTAAAAGAAACCCCCATCTATTTAGTTATTTGTTTAGTCACTTATAACTTTAACAGATGGGGTTTTTCTGTGCAACCAATTTTAAGGGTTTTCAATACTTTAAAACACATACATACCAACACTTCAACGCACCTTTCAGCAACTAAAATAAAAATGACGTTATTTCTATATGTATCAAGATAAGAAAGAACAAGTTCAAAACCATCAAAAAAAGACACCTTTTCAGGTGCTTTTTTTATTTTATAAACTCATTCCCTGATCTCGACTTCGTTCTTTTTTTACCTCTCGGTTATGAGTTAGTTCAAATTCGTTCTTTTTAGGTTCTAAATCGTGTTTTTCTTGGAATTGTGCTGTTTTATCCTTTACCTTGTCTACAAACCCCTTAAAAACGTTTTTAAAGGCTTTTAAGCCGTCTGTACGTTCCTTAAGGAATTCCTTAGTGCTTTCATAGATTAAACTCACATCACGCTTTAAATCGCTTATTTTAGACTTTAAAGACTTGTTTTCTTCAAGCAACTCATTATAATCATTTACATTTTCATTAAATCGCTCTACAAGACCACTATATTTTTCTTTAACTTGCCCATGTTCTTTACTTAATTTTTTATATTCTCTCGCCATATCAGTACTCATGAGATTTCTAACATGCTGTTTTAACCTATCGTTATCTCTCGCAGCAGTCACTAAGTTTTTATAATCACGCTCCGATATAACAACATTTTTGGTTGGTTTCTTTTCTGTTTTCATTATTTCTTTTCCCAAACCAAACATGGACTTTTCACCCGTTGGCACTTCAACACTTTTCATGTGTCGTTTCGCTGGTACTTCTAAATCTGATTTAACTTTATCGCTATAAGCAGTCCATTCATCTTTTTTAACTGCTAAATTTTTTTCTAGAAAATCAATCTCTTTTTCCAAAGTTTGTTTTTTAAATTTAGCTGTCTCAATATGTTTACGGTCAGAGCCACGTTCACCACGCTTCAACTCAAAACCCTGTTTTTTCATATGCTCGGGGAATTTATCTTGTAGCCATAACAGTTCTTGACGATTAAACACATTTTTTCCTTGCAGTTTTCCATCACGCATAGGCACAACACCTAAATGCATGTGAGGGGTTTGCTCATCATTATGAACTGTTGCATAAGCAATATTTTGCTTGCCATATCGTTCGGAAAATAATTTATAACTTTCCTCTCAAAATCGTTTTTGTTCTCCT

**β-Gal-split-GFP expression plasmid (pUB110-gal-split-gfp) sequence**

The PamyQ' promoter is coloured purple andβ-gal is lighted orange. split-gfp11 and split-gfp1-10 are labeled in green and the underlined portion is the linker. RBS is marked in gray.

GGATCGGCGGCGTTCTGTTTCTGCTTCGGTATGTGATTGTGAAGCTGGCTTACAGAAGAGCGGTAAAAGAAGAAATAAAAAAGAAATCATCTTTTTTGTTTGGAAAGCGAGGGAAGCGTTCACAGTTTCGGGCAGCTTTTTTTATAGGAACATTGATTTGTATTCACTCTGCCAAGTTGTTTTGATAGAGTGATTGTGATAATTTTAATGTAAGCGATAACAAAATTCTCCAGTCTTCACATCGGTTTGAAAGGAGGAAGCGGAAGAATGAAGTAAGAGGGATTTTTGACTCCGAAGTAAGTCTTCAAAAAATCAAATAAGGAGTGTCAAGAATGCGTCGTATTAACTTTAACGATAACTGGCGTTTTCAGCGCGAGATTAGTACCAGTTTACGCGAAGCACAGAAGCCCAGCTTCAATGACCACTCTTGGCGTCAATTAAGTTTGCCGCACGACTGGTCTATCGAGCTGGACTTTAACAAGGATTCATTAGCCACTCATGAAGGTGGATACTTAGATGGAGGAGTTGGGTGGTATCGCAAAACGTTCACAGTCCCATCGGCAATGGAGGGTAAGCGCATCTCCTTAGATTTTGATGGTGTTTATATGAACTCAACTACTTATCTGAACGGAGAAGAGCTTGGCACGTATCCATTCGGGTATAATGCGTTCAGCTACGATATCACTGATAAATTGTTCATGGACGGCCGTGAGAACGTGCTGGCGGTCAAGGTAGATAACACCCAACCATCTTCTCGTTGGTATAGTGGGAGTGGGATCTATCGCAACGTCTACCTGACTGTAACGAACCCTGTCCATGTCGCACGCTATGGCACCTTTGTAACCACCCCGGACTTGGAGAGTGCATACGCGGCCCGCAAGGCTGAAGTCAATATTAAGACTAAAATCAACAACGATAGTGATGCAGCGGTTCAAGTTAAGGTCAAAAGCACGATTTATGATACAGATGGTAAAGAAGTTGCGAGTGTCGTTTCGCAGGAAAAAACAGCGGCAGCGGGCACAACTGCGCATTTCGAAGACAACACGGTCATTGAAAATCCCGAGTTGTGGAGTTTAGATAATCCTTACCGTTACAAATTGGTGACGGACGTGTTAATTGGGGGCGAGACAGTAGACACTTATGAAACACGCTTCGGGGCACGCTTCTTCAAATTCGATGCTAACGAGGGGTTTAGTTTAAATGGAAAGCCAATGAAGCTGTACGGAGTAAGTATGCACCACGACTTGGGAGCCCTGGGGGCAGCCACGAACGCACGTGCTGTTGAGCGTCAACTTCAGATTATGAAGGATATGGGAGTAAATGCAATTCGTGGTACGCACAATCCGGTATCCCCAGAGTTCCTTGAAGCCGTTAATAATTTGGGGTTACTGCTGATCGAAGAGGCTTTTGATTGCTGGTCGCAGTCCAAAAAAACCTATGACTATGGCCGTTTCTTTACACGTTGGGCCGAACACGATGTGAAAGAGATGGTTGACCGCGGCAAAAATGAACCAAGTATCATCATGTGGTCGATTGGCAATGAAATCTACGACACCACATCCCCATCTGGAGTGGAAACCGCTCGCAACTTAGTACGTTGGATCAAAGAGATTGATACGACGCGCCCGACAACCATTGGAGAAGATAAGACCCGTGGCGACAAAGTCAATGTGACACCGATTGACCCAAACATTTTAGAGATTTTCCATACAGTAGATGTAGTTGGATTAAATTACAGCGAGAATAATTATGTGGGATACCACGAACAGCACCCTAACTGGAAGTTATACGGCTCAGAGACGTCGAGTGCTACTCGCTCTCGCGGTGTATATACACACCCCTACGAGTATAACCTGGGGACTAAGTATGACGACTTGCAACAAAGCTCCTACGATAATGACTACGTTCCGTGGGGACGTACAGCAGAAGATGCGTGGAAGTCAGATCGTGATTTGAAGCACTTTGCAGGCCAGTTTATTTGGACTGGCTTTGACTACATTGGAGAACCTACACCATATTATGACAGCTACCCAGCCAAGAGTTCGTACTTTGGGGCCGTTGATACAGCGGGCTTTCCTAAGGATATTTTCTACTACTATCAATCGCAGTGGAAAAAAGAACCTATGGTACACTTATTGCCCCATTGGAATTGGACTGAGGGTGAACCCGTGCGCGTATTAGCTTATACTAACGCGCATCAGGTAGAACTGTTCCTGAATGGCAAGAGTCTTGGTGTCCGCGGATATGAGAATAAGAAAACATCTTGGGGTGCTCCCTATAAGGAAACTAAGGACGGTAAAACTTACCTGGAATGGGCAGTGCCATTCAAGGCTGGGACTTTGGAGGCAGTTGCGATGGATGAAAACGGTAAGGAAATCGCGCGTGACCAAGTCACGACAGCCGGGGCACCGGCCGCGGTAAAGTTAACAGCTGATCGCAAAGTCATTAAGGCCGACGGAACCGATTTATCTTTTATCACAGCAGAGATCGTAGATAGTAAGGGCAATGTTGTCCCCAATGCTGATCACTTAATTCAGTTTCATTTGTCAGGCCACGGGGAGTTGGCCGGTGTAGACAATGGTGATGCCGCAAGCGTCGAGCGCTATAAGGACAATAAACGTAAGGCTTTTAGCGGTAAGGCTTTGGCGATTGTACAGTCGAATAAATTGGATGGTAATATTACTTTGCACGCCTCGGCAGAGGGGCTTTCTAGTGGCAACGTAACAATCTTTACGACGGCCTCTGCTGATCAGGGCGGAGGCTCTGGCGGAGGATCTCGCGATCACATGGTTCTTCATGAATATGTTAATGCAGCAGGCATCACATGAAAGGAGTGTCAAGAATGTCTAAAGGCGAAGAACTGTTTACGGGCGTTGTTCCTATTCTTGTTGAACTTGATGGAGATGTTAATGGACATAAATTTAGCGTTAGAGGAGAAGGAGAAGGCGATGCAACGATCGGCAAACTGACGCTGAAATTTATCTGCACGACGGGAAAACTCCCAGTTCCGTGGCCTACGCTGGTTACGACACTTACGTATGGAGTTCAATGCTTTTCTCGCTATCCGGATCACATGAAACGCCATGATTTTTTTAAATCTGCAATGCCGGAAGGCTATGTTCAAGAACGCACAATCAGCTTTAAAGATGATGGCAAATATAAAACGCGCGCAGTTGTTAAATTTGAAGGCGATACACTGGTTAATCGCATCGAACTGAAAGGCACGGATTTTAAAGAAGATGGCAATATCCTGGGCCATAAACTGGAATATAATTTTAATAGCCATAATGTTTATATTACAGCAGATAAACAAAAAAATGGCATCAAAGCAAATTTTACAGTTAGACATAATGTTGAAGATGGCTCAGTTCAACTTGCAGATCATTATCAACAAAATACACCTATTGGCGATGGACCCGTTCTGCTGCCTGATAATCATTATCTTAGCACACAAACAGTTCTGTCTAAAGATCCGAATGAAAAATAAGGTAATAAAAAAACACCTCCAAGCTGAGTGCGGGTATCAGCTTGGAGGTGCGTTTATTTTTTCAGCCGTATGACAAGGTCGGCATCAGGTGTGACAAATACGGTATGCTGGCTGTCATAGGTGACAAATCCGGGTTTTGCGCCGTTTGGCTTTTTCACATGTCTGATTTTTGTATAATCAACAGGCACGGAGCCGGAATCTTTCGCCTTGGAAAAATAAGCGGCGATCGTAGCTGCTTCCAATATGGATTGTTCATCGGGATCGCTGCTTTTAATCACAACGTGGGAGATCCAGTTGCTCAAAAAAATCTCGGTCAGATGTTACTAGCAACTCATTTACAAGAACAGCATCTTTCCTCGTTTTTCTTGTACCTGTTTTTTGTGATTCAATAATTTCTTTGACACGTTCGTTGTAATCAATATTTTTATCATTTTTCAAATCATAATTTTCACGTGTTCGCTCATGGTCAATATCATCATTCGTTCTACTTTTTCGCTCTCTTTGATTATGAAATTGCATGCCTTTTAGTCCAGCTGATTTCACTTTTTGCATTCTACAAACTGCATAACTCATATGTAAATCGCTCCTTTTTAGGTGGCACAAATGTGAGGCATTTTCGCTCTTTCCGGCAACCACTTCCAAGTAAAGTATAACACACTATACTTTATATTCATAAAGTGTGTGCTCTGCGAGGCGCGGCAGTGCCGACCAAAACCATAAAACCTTTAAGACCTTTCTTTTTTTTACGAGAAAAAAGAAACAAAAAAACCTGCCCTCTGCCACCTCAGCAAAGGGGGGTTTTGCTCTCGTGCTCGTTTAAAAATCAGCAAGGGACAGGTAGTATTTTTTGAGAAGATCACTCAAAAAATCTCCACCTTTAAACCCTTGCCAATTTTTATTTTGTCCGTTTTGTCTAGCTTACCGAAAGCCAGACTCAGCAAGAATAAAATTTTTATTGTCTTTCGGTTTTCTAGTGTAACGGACAAAACCACTCAAAATAAAAAAGATACAAGAGAGGTCTCTCGTATCTTTTATTCAGCAATCGCGCCCGATTGCTGAACAGATTAATAATAGATTTTAGCTTTTTATTTGTTGAAAAAAGCTAATCAAATTGTTGTCGGGATCAATTACTGCAAAGTCTCGTTCATCCCACCACTGATCTTTTAATGATGTATTGGGGTGCAAAATGCCCAAAGGCTTAATATGTTGATATAATTCATCAATTCCCTCTACTTCAATGCGGCAACTAGCAGTACCAGCAATAAACGACTCCGCACCTGTACAAACCGGTGAATCATTACTACGAGGCGCCAGCCTTCATCACTTGCCTCCCATAGATGAATCCGAACCTCATTACACATTAGAACTGCGAATCCATCTTCATGGTGAACCAAAGTGAAACCTACTTTATCCCAATAAAAACCTATACTCTTTTTAATATCCCCGACTGGCAATGCCGGGATAGACTGTAACATTCTCACGCATAAAATCCCCTTTCATTTTCTAATGTAAATCTATTACCTTATTATTAATTCAATTCGCTCATAATTAATCCTTTTTCTTATTACGCAAAATGGCCCGATTTAAGCACACCCTTTATTCCGTTAATGCGCCATGACAGCCATGATAATTACTAATACTAGGAGAAGTTAATAAATACGTAACCAACATGATTAACAATTATTAGAGGTCATCGTTCAAAATGGTATGCGTTTTGACACATCCACTATATATCCGTGTCGTTCTGTCCACTCCTGAATCCCATTCCAGAAATTCTCTAGCGATTCCAGAAGTTTCTCAGAGTCGGAAAGTTGACCAGACATTACGAACTGGCACAGATGGTCATAACCTGAAGGAAGATCTGATTGCTTAACTGCTTCAGTTAAGACCGAAGCGCTCGTCGTATAACAGATGCGATGATGCAGACCAATCAACATGGCACCTGCCATTGCTACCTGTACAGTCAAGGATGGTAGAAATGTTGTCGGTCCTTGCACACGAATATTACGCCATTTGCCTGCATATTCAAACAGCTCTTCTACGATAAGGGCACAAATCGCATCGTGGAACGTTTGGGCTTCTACCGATTTAGCAGTTTGATACACTTTCTCTAAGTATCCACCTGAATCATAAATCGGCAAAATAGAGAAAAATTGACCATGTGTAAGCGGCCAATCTGATTCCACCTGAGATGCATAATCTAGTAGAATCTCTTCGCTATCAAAATTCACTTCCACCTTCCACTCACCGGTTGTCCATTCATGGCTGAACTCTGCTTCCTCTGTTGACATGACACACATCATCTCAATATCCGAATAGGGCCCATCAGTCTGACGACCAAGAGAGCCATAAACACCAATAGCCTTAACATCATCCCCATATTTATCCAATATTCGTTCCTTAATTTCATGAACAATCTTCATTCTTTCTTCTCTAGTCATTATTATTGGTCCATTCACTATTCTCATTCCCTTTTCAGATAATTTTAGATTTGCTTTTCTAAATAAGAATATTTGGAGAGCACCGTTCTTATTCAGCTATTAATAACTCGTCTTCCTAAGCATCCTTCAATCCTTTTAATAACAATTATAGCATCTAATCTTCAACAAACTGGCCCGTTTGTTGAACTACTCTTTAATAAAATAATTTTTCCGTTCCCAATTCCACATTGCAATAATAGAAAATCCATCTTCATCGGCTTTTTCGTCATCATCTGTATGAATCAAATCGCCTTCTTCTGTGTCATCAAGGTTTAATTTTTTATGTATTTCTTTTAACAAACCACCATAGGAGATTAACCTTTTACGGTGTAAACCTTCCTCCAAATCAGACAAACGTTTCAAATTCTTTTCTTCATCATCGGTCATAAAATCCGTATCCTTTACAGGATATTTTGCAGTTTCGTCAATTGCCGATTGTATATCCGATTTATATTTATTTTTCGGTCGAATCATTTGAACTTTTACATTTGGATCATAGTCTAATTTCATTGCCTTTTTCCAAAATTGAATCCATTGTTTTTGATTCACGTAGTTTTCTGTATTCTTAAAATAAGTTGGTTCCACACATACCAATACATGCATGTGCTGATTATAAGAATTATCTTTATTATTTATTGTCACTTCCGTTGCACGCATAAAACCAACAAGATTTTTATTAATTTTTTTATATTGCATCATTCGGCGAAATCCTTGAGCCATATCTGACAAACTCTTATTTAATTCTTCGCCATCATAAACATTTTTAACTGTTAATGTGAGAAACAACCAACGAACTGTTGGCTTTTGTTTAATAACTTCAGCAACAACCTTTTGTGACTGAATGCCATGTTTCATTGCTCTCCTCCAGTTGCACATTGGACAAAGCCTGGATTTACAAAACCACACTCGATACAACTTTCTTTCGCCTGTTTCACGATTTTGTTTATACTCTAATATTTCAGCACAATCTTTTACTCTTTCAGCCTTTTTAAATTCAAGAATATGCAGAAGTTCAAAGTAATCAACATTAGCGATTTTCTTTTCTCTCCATGGTCTCACTTTTCCACTTTTTGTCTTGTCCACTAAAACCCTTGATTTTTCATCTGAATAAATGCTACTATTAGGACACATAATATTAAAAGAAACCCCCATCTATTTAGTTATTTGTTTAGTCACTTATAACTTTAACAGATGGGGTTTTTCTGTGCAACCAATTTTAAGGGTTTTCAATACTTTAAAACACATACATACCAACACTTCAACGCACCTTTCAGCAACTAAAATAAAAATGACGTTATTTCTATATGTATCAAGATAAGAAAGAACAAGTTCAAAACCATCAAAAAAAGACACCTTTTCAGGTGCTTTTTTTATTTTATAAACTCATTCCCTGATCTCGACTTCGTTCTTTTTTTACCTCTCGGTTATGAGTTAGTTCAAATTCGTTCTTTTTAGGTTCTAAATCGTGTTTTTCTTGGAATTGTGCTGTTTTATCCTTTACCTTGTCTACAAACCCCTTAAAAACGTTTTTAAAGGCTTTTAAGCCGTCTGTACGTTCCTTAAGGAATTCCTTAGTGCTTTCATAGATTAAACTCACATCACGCTTTAAATCGCTTATTTTAGACTTTAAAGACTTGTTTTCTTCAAGCAACTCATTATAATCATTTACATTTTCATTAAATCGCTCTACAAGACCACTATATTTTTCTTTAACTTGCCCATGTTCTTTACTTAATTTTTTATATTCTCTCGCCATATCAGTACTCATGAGATTTCTAACATGCTGTTTTAACCTATCGTTATCTCTCGCAGCAGTCACTAAGTTTTTATAATCACGCTCCGATATAACAACATTTTTGGTTGGTTTCTTTTCTGTTTTCATTATTTCTTTTCCCAAACCAAACATGGACTTTTCACCCGTTGGCACTTCAACACTTTTCATGTGTCGTTTCGCTGGTACTTCTAAATCTGATTTAACTTTATCGCTATAAGCAGTCCATTCATCTTTTTTAACTGCTAAATTTTTTTCTAGAAAATCAATCTCTTTTTCCAAAGTTTGTTTTTTAAATTTAGCTGTCTCAATATGTTTACGGTCAGAGCCACGTTCACCACGCTTCAACTCAAAACCCTGTTTTTTCATATGCTCGGGGAATTTATCTTGTAGCCATAACAGTTCTTGACGATTAAACACATTTTTTCCTTGCAGTTTTCCATCACGCATAGGCACAACACCTAAATGCATGTGAGGGGTTTGCTCATCATTATGAACTGTTGCATAAGCAATATTTTGCTTGCCATATCGTTCGGAAAATAATTTATAACTTTCCTCTCAAAATCGTTTTTGTTCTCCT

**Plasmids for sgRNA expression**

**sgRNA expression plasmid (pAD123-sgRNA) sequence**

The P43 promoter is coloured purple and N20 is lighted yellow. sgRNA handle is coloured blue.

GAATTCGAGCTCGGTACCCGGGGATCCTCTAGATTTAAGAAGGAGATATACATATTTTACATTTTTAGAAATGGGCGTGAAAAAAAGCGCGCGATTATGTAAAATATAANNNNNNNNNNNNNNNNNNNNGTTTTAGAGCTAGAAATAGCAAGTTAAAATAAGGCTAGTCCGTTATCAACTTGAAAAAGTGGCACCGAGTCGGTGCATCTCCTGCAGGCATGCAAGCTTGAGTAGGACAAATCCGCCGAGCTTCGACGAGATTTTCAGGAGCTAAGGAAGCTAAAATGGAGAAAAAAATCACTGGATATACCACCGTTGATATATCCCAATGGCATCGTAAAGAACATTTTGAGGCATTTCAGTCAGTTGCTCAATGTACCTATAACCAGACCGTTCAGAACAAAGAATACAAGAAAATATTTACAAAAAATCAATTTAACAATTCCTTAAAACATGCAGGAATTGACGATTTAAACAATATTAGCTTTGAACAATTCTTATCTCTTTTCAATAGCTATAAATTATTTAATAAGTAAGTTAAGGGATGCATAAACTGCATCCCTTAACTTGTTTTTCGTGTGCCTATTTTTTGTGAATCGCTAAGAAACCATTATTATCATGACATTAACCTATAAAAATAGGCGTATCACGAGGCCCTTTCGTCTCGCGCGTTTCGGTGATGACGGTGAAAACCTCTGACACATGCAGCTCCCGGAGACGGTCACAGCTTGTCTGTAAGCGGATGCCGGGAGCAGACAAGCCCGTCAGGGCGCGTCAGCGGGTGTTGGCGGGTGTCGGGGCTGGCTTAACTATGCGGCATCAGAGCAGATTGTACTGAGAGTGCACCATACAAAACATATTTCAACACAATACAAATGGGTTAGTTAAAAAAGCAGGCCTTCTAAAGGTCTGCTTTTTTTATTTGATTATGTAATTTTTAATGCCAGGATGCCAATAAGCCATAACCTCAAATGCACCATTTGCAACCTCGTCATCTTCTTCCTCAATCTTGACCAGATCGCCGTCCTCCGCATCACCAAGATTCAGCTCTTTATGTATCTCCTTCAAAATGCCACCGTATCCAATTAACCTTCGAGCTGCCAACGCATCATCCAAGTAAAGCACCGTGTTCAGATTGTCTTCAGTCACCTTATTACCGCGCACACAATCCGTATCCTTAACCGGATATTTAGAGATTTCGCGAACAGCTTTTTGCTCCATCATTGCGTTCCGCACATCGTTTTCAATCTGTTCAGCGTCAATCTTAGCTTTACCTTTCACTCGACGAATATCGACAATTGGAGTGTAATCCAATTTCATCGCCTTTTTCCAAAGGCTCGTCCACTCCGCCTGCTTAATATAGTTTTTCCCAAAATAATTTTTCCTTACTGGTATCAACACATGAAAATGAGGATGATATGTATCTTCTTCATGATTTTTGGTAATCTCTAAAGCTCTGAAAAATCCAAGAACCGAAGTTTTTACTTTTTTGTACTGGAACAGTTTCCTAAAGCCTTCCATCATCGCAGAAATTTGTGGCTTCAGCCGTTCTCCCTTTACATTTCGAATCGTCAGCGTGAGAAAAATCCATCCGCAGCCGTACTGTCTATTGGCTTCCTCTACGATCAACTTATTGTGATAAGCAATTTTTAACGACCTGCGCCACGCACACATCGGACATAACCTCACTTTACAAAAATGGGCTTGATACAGTTTTAACTTGCCCGTCTCCGGGTCTCTCTTAAACGAAAGATACTCTGCACAACTAATTAGTTTTTCAGCCTTTTTGCCATAGTAAGGTGCCCCAATCTTACTCTCTAACGCTTCGTAATGCTCCGCCATGAGGTTCGTCCGTCTCTTTTTCCCCTTCCAATCCCGCTTTTTACCTGTTGCGGTTTTATCTTCGAGGATGCTATAATCATTTTCAGATGAATAAATCAACAAAAAAACTCCTTCTGAGCTAGTTCTCTAGCATTCTATTATTTTGATTCGACACCTTAATAATAGCAGAAGGAGTTTTTACCTGTCAAAGAACCATCAAACCCTTGATACACAAGGCTTTGACCTAATTTTGAAAAATGATGTTGTTTCTATATAGTATCAAGATAAGAAAGAAAAGGATTTTTCGCTACGCTCAAATCCTTTAAAAAAACACAAAAGACCACATTTTTTAATGTGGTCTTTATTCTTCAACTAAAGCACCCATTAGTTCAACAAACGAAAATTGGATAAAGTGGGATATTTTTAAAATATATATTTATGTTACAGTAATATTGACTTTTAAAAAAGGATTGATTCTAATGAAGAAAGCAGACAAGTAAGCCTCCTAAATTCACTTTAGATAAAAATTTAGGAGGCATATCAAATGAACTTTAATAAAATTGATTTAGACAATTGGAAGAGAAAAGAGATATTTAATCATTATTTGAACCAACAAACGACTTTTAGTATAACCACAGAAATTGATATTAGTGTTTTATACCGAAACATAAAACAAGAAGGATATAAATTTTACCCTGCATTTATTTTCTTAGTGACAAGGGTGATAAACTCAAATACAGCTTTTAGAACTGGTTACAATAGCGACGGAGAGTTAGGTTATTGGGATAAGTTAGAGCCACTTTATACAATTTTTGATGGTGTATCTAAAACATTCTCTGGTATTTGGACTCCTGTAAAGAATGACTTCAAAGAGTTTTATGATTTATACCTTTCTGATGTAGAGAAATATAATGGTTCGGGGAAATTGTTTCCCAAAACACCTATACCTGAAAATGCTTTTTCTCTTTCTATTATTCCATGGACTTCATTTACTGGGTTTAACTTAAATATCAATAATAATAGTAATTACCTTCTACCCATTATTACAGCAGGAAAATTCATTAATAAAGGTAATTCAATATATTTACCGCTATCTTTACAGGTACATCATTCTGTTTGTGATGGTTATCATGCAGGATTGTTTATGAACTCTATTCAGGAATTGTCAGATAGGCCTAATGACTGGCTTTTATAATATGAGATAATGCCGACTGTACTTTTTACAGTCGGTTTTCTAATGTCACTAACCTGCCCCGTTAGTCGCCATTCGCCAGCTGCCTCGCGCGTTTCGGTGATGACGGTGAAAACCTCTGACACATGCAGCTCCCGGAGACGGTCACAGCTTGTCTGTAAGCGGATGCCGGGAGCAGACAAGCCCGTCAGGGCGCGTCAGCGGGTGTTGGCGGGTGTCGGGGCGCAGCCATGACCCAGTCACGTAGCGATAGCGGAGTGTATACTGGCTTAACTATGCGGCATCAGAGCAGATTGTACTGAGAGTGCACCATATGCGGTGTGAAATACCGCACAGATGCGTAAGGAGAAAATACCGCATCAGGCGCTCTTCCGCTTCCTCGCTCACTGACTCGCTGCGCTCGGTCGTTCGGCTGCGGCGAGCGGTATCAGCTCACTCAAAGGCGGTAATACGGTTATCCACAGAATCAGGGGATAACGCAGGAAAGAACATGTGAGCAAAAGGCCAGCAAAAGGCCAGGAACCGTAAAAAGGCCGCGTTGCTGGCGTTTTTCCATAGGCTCCGCCCCCCTGACGAGCATCACAAAAATCGACGCTCAAGTCAGAGGTGGCGAAACCCGACAGGACTATAAAGATACCAGGCGTTTCCCCCTGGAAGCTCCCTCGTGCGCTCTCCTGTTCCGACCCTGCCGCTTACCGGATACCTGTCCGCCTTTCTCCCTTCGGGAAGCGTGGCGCTTTCTCAATGCTCACGCTGTAGGTATCTCAGTTCGGTGTAGGTCGTTCGCTCCAAGCTGGGCTGTGTGCACGAACCCCCCGTTCAGCCCGACCGCTGCGCCTTATCCGGTAACTATCGTCTTGAGTCCAACCCGGTAAGACACGACTTATCGCCACTGGCAGCAGCCACTGGTAACAGGATTAGCAGAGCGAGGTATGTAGGCGGTGCTACAGAGTTCTTGAAGTGGTGGCCTAACTACGGCTACACTAGAAGGACAGTATTTGGTATCTGCGCTCTGCTGAAGCCAGTTACCTTCGGAAAAAGAGTTGGTAGCTCTTGATCCGGCAAACAAACCACCGCTGGTAGCGGTGGTTTTTTTGTTTGCAAGCAGCAGATTACGCGCAGAAAAAAAGGATCTCAAGAAGATCCTTTGATCTTTTCTACGGGGTCTGACGCTCAGTGGAACGAAAACTCACGTTAAGGGATTTTGGTCATGAGATTATCAAAAAGGATCTTCACCTAGATCCTTTTAAATTAAAAATGAAGTTTTAAATCAATCTAAAGTATATATGAGTAAACTTGGTCTGACAGTTACCAATGCTTAATCAGTGAGGCACCTATCTCAGCGATCTGTCTATTTCGTTCATCCATAGTTGCCTGACTCCCCGTCGTGTAGATAACTACGATACGGGAGGGCTTACCATCTGGCCCCAGTGCTGCAATGATACCGCGAGACCCACGCTCACCGGCTCCAGATTTATCAGCAATAAACCAGCCAGCCGGAAGGGCCGAGCGCAGAAGTGGTCCTGCAACTTTATCCGCCTCCATCCAGTCTATTAATTGTTGCCGGGAAGCTAGAGTAAGTAGTTCGCCAGTTAATAGTTTGCGCAACGTTGTTGCCATTGCTGCAGGCATCGTGGTGTCACGCTCGTCGTTTGGTATGGCTTCATTCAGCTCCGGTTCCCAACGATCAAGGCGAGTTACATGATCCCCCATGTTGTGCAAAAAAGCGGTTAGCTCCTTCGGTCCTCCGATCGTTGTCAGAAGTAAGTTGGCCGCAGTGTTATCACTCATGGTTATGGCAGCACTGCATAATTCTCTTACTGTCATGCCATCCGTAAGATGCTTTTCTGTGACTGGTGAGTACTCAACCAAGTCATTCTGAGAATAGTGTATGCGGCGACCGAGTTGCTCTTGCCCGGCGTCAACACGGGATAATACCGCGCCACATAGCAGAACTTTAAAAGTGCTCATCATTGGAAAACGTTCTTCGGGGCGAAAACTCTCAAGGATCTTACCGCTGTTGAGATCCAGTTCGATGTAACCCACTCGTGCACCCAACTGATCTTCAGCATCTTTTACTTTCACCAGCGTTTCTGGGTGAGCAAAAACAGGAAGGCAAAATGCCGCAAAAAAGGGAATAAGGGCGACACGGAAATGTTGAATACTCATACTCTTCCTTTTTCAATATTATTGAAGCATTTATCAGGGTTATTGTCTCATGAGCGGATACATATTTGAATGTATTTAGAAAAATAAACAAATAGGGGTTCCGCGCACATTTCCCCGAAAAGTGCCACCTGACGTCTAAGAAACCATTATTATCATGACATTAACCTATAAAAATAGGCGTATCACGAGGCCCTTTCGTCTTCAA

**sgRNA integration plasmid (pET-sgRNA) sequence**

The P43 promoter is coloured purple and N20 is lighted yellow. sgRNA handle is coloured blue. Homology arm regions are underlined. Cre-lox sequences are marked in gray.

GTGATGTCAAAGCTTGAAAAAACGCACGTAACAAAAGCAAAATTTATGCTCCATGGGGGAGACTACAACCCCGATCAGTGGCTGGATCGGCCCGATATTTTAGCTGACGATATCAAACTGATGAAGCTTTCTCATACGAATACGTTTTCTGTCGGCATTTTTGCATGGAGCGCACTTGAGCCGGAGGAGGGCGTATATCAATTTGAATGGCTGGATGATATTTTTGAGCGGATTCACAGTATAGGCGGCCGGGTCATATTAGCAACGCCGAGCGGAGCCCGTCCGGCCTGGCTGTCGCAAACCTATCCGGAAGTTTTGCGCGTCAATGCCTCCCGCGTCAAACAGCTGCACGGCGGAAGGCACAACCACTGCCTCACATCTAAAGTCTACCGAGAAAAAACACGGCACATCAACCGCTTATTAGCAGAACGATACGGACATCACCCGGCGCTGTTAATGTGGCACATTTCAAACGAATACGGGGGAGATTGCCACTGTGAATTTTACATTTTTAGAAATGGGCGTGAAAAAAAGCGCGCGATTATGTAAAATATAANNNNNNNNNNNNNNNNNNNNGTTTTAGAGCTAGAAATAGCAAGTTAAAATAAGGCTAGTCCGTTATCAACTTGAAAAAGTGGCACCGAGTCGGTGCGGTAATAAAAAAACACCTCCAAGCTGAGTGCGGGTATCAGCTTGGAGGTGCGTTTATTTTTTCAGCCGTATGACAAGGTCGGCATCAGGTGTGACAAATACGGTATGCTGGCTGTCATAGGTGACAAATCCGGGTTTTGCGCCGTTTGGCTTTTTCACATGTCTGATTTTTGTATAATCAACAGGCACGGAGCCGGAATCTTTCGCCTTGGAAAAATAAGCGGCGATCGTAGCTGCTTCCAATATGGATTGTTCATCGGGATCGCTGCTTTTAATCACAACGTGGGATACCGTTCGGTATAGCATACATTATACGAAGTTATTTCAACAAACGGGCCATATTGTTGTATAAGTGATGAAATACTGAATTTAAAACTTAGTTTATATGTGGTAAAATGTTTTAATCAAGTTTAGGAGGAATTAATTATGAAGTGTAATGAATAATGAATGTAACAGGGTTCAATTAAAAGAGGGAAGCGTATCATTAACCCTATAAACTACGTCTGCCCTCATTATTGGAGGGTGAAATGTGAATACATCCTATTCACAATCGAATTTACGACACAACCAAATTTTAATTTGGCTTTGCATTTTATCTTTTTTTAGCGTATTAAATGAAATGGTTTTGAACGTCTCATTACCTGATATTGCAAATGATTTTAATAAACCACCTGCGAGTACAAACTGGGTGAACACAGCCTTTATGTTAACCTTTTCCATTGGAACAGCTGTATATGGAAAGCTATCTGATCAATTAGGCATCAAAAGGTTACTCCTATTTGGAATTATAATAAATTGTTTCGGGTCGGTAATTGGGTTTGTTGGCCATTCTTTCTTTTCCTTACTTATTATGGCTCGTTTTATTCAAGGGGCTGGTGCAGCTGCATTTCCAGCACTCGTAATGGTTGTAGTTGCGCGCTATATTCCAAAGGAAAATAGGGGTAAAGCATTTGGTCTTATTGGATCGATAGTAGCCATGGGAGAAGGAGTCGGTCCAGCGATTGGTGGAATGATAGCCCATTATATTCATTGGTCCTATCTTCTACTCATTCCTATGATAACAATTATCACTGTTCCGTTTCTTATGAAATTATTAAAGAAAGAAGTAAGGATAAAAGGTCATTTTGATATCAAAGGAATTATACTAATGTCTGTAGGCATTGTATTTTTTATGTTGTTTACAACATCATATAGCATTTCTTTTCTTATCGTTAGCGTGCTGTCATTCCTGATATTTGTAAAACATATCAGGAAAGTAACAGATCCTTTTGTTGATCCCGGATTAGGGAAAAATATACCTTTTATGATTGGAGTTCTTTGTGGGGGAATTATATTTGGAACAGTAGCAGGGTTTGTCTCTATGGTTCCTTATATGATGAAAGATGTTCACCAGCTAAGTACTGCCGAAATCGGAAGTGTAATTATTTTCCCTGGAACAATGAGTGTCATTATTTTCGGCTACATTGGTGGGATACTTGTTGATAGAAGAGGTCCTTTATACGTGTTAAACATCGGAGTTACATTTCTTTCTGTTAGCTTTTTAACTGCTTCCTTTCTTTTAGAAACAACATCATGGTTCATGACAATTATAATCGTATTTGTTTTAGGTGGGCTTTCGTTCACCAAAACAGTTATATCAACAATTGTTTCAAGTAGCTTGAAACAGCAGGAAGCTGGTGCTGGAATGAGTTTGCTTAACTTTACCAGCTTTTTATCAGAGGGAACAGGTATTGCAATTGTAGGTGGTTTATTATCCATACCCTTACTTGATCAAAGGTTGTTACCTATGGAAGTTGATCAGTCAACTTATCTGTATAGTAATTTGTTATTACTTTTTTCAGGAATCATTGTCATTAGTTGGCTGGTTACCTTGAATGTATATAAACATTCTCAAAGGGATTTCTAAATCGTTAAGGGATCAACTTTGGGAGAGAGTTCAAAATTGATCCTTTTTTTATAACAGGAATTCCCGGGGATCCGTCGACCTGCAGATCTCTAGAAGCTTATAACTTCGTATAGCATACATTATACGAACGGTATTCAAGCTATATTTGGAGTTGAGCCTCTTGAAACGGACACCCTGTATCCGAAGGATCGAAACGCTGTCAGCTACCGCAGCCAAATATATGAAATGAAGGATTATGCAACCGTGATTGATGTAAAGACAGCTTCAGTGGAAGCGGTGTATCAAGAAGATTTTTATGCGCGCACGCCAGCGGTCACAAGCCATGAGTATCAGCAGGGCAAGGCGTATTTTATCGGCGCGCGTTTGGAGGATCAATTTCAGCGTGATTTCTATGAGGGTCTGATCACAGACCTGTCTCTCTCTCCAGTTTTTCCGGTTCGGCACGGAAAAGGCGTCTCCGTACAAGCGAGGCAGGATCAGGACAATGATTATATTTTTGTCATGAATTTCACGGAAGAAAAACAGCTGGTCACGTTTGATCAGAGTGTGAAGGACATAATGACAGGAGACATATTGTCAGGCGACCTGACGATGGAAAAGTATGAAGTGAGAATTGTCGTAAACACACATTAGGCGCAACGCAATTAATGTAAGTTAGCTCACTCATTAGGCACCGGGATCTCGACCGATGCCCTTGAGAGCCTTCAACCCAGTCAGCTCCTTCCGGTGGGCGCGGGGCATGACTATCGTCGCCGCACTTATGACTGTCTTCTTTATCATGCAACTCGTAGGACAGGTGCCGGCAGCGCTCTGGGTCATTTTCGGCGAGGACCGCTTTCGCTGGAGCGCGACGATGATCGGCCTGTCGCTTGCGGTATTCGGAATCTTGCACGCCCTCGCTCAAGCCTTCGTCACTGGTCCCGCCACCAAACGTTTCGGCGAGAAGCAGGCCATTATCGCCGGCATGGCGGCCCCACGGGTGCGCATGATCGTGCTCCTGTCGTTGAGGACCCGGCTAGGCTGGCGGGGTTGCCTTACTGGTTAGCAGAATGAATCACCGATACGCGAGCGAACGTGAAGCGACTGCTGCTGCAAAACGTCTGCGACCTGAGCAACAACATGAATGGTCTTCGGTTTCCGTGTTTCGTAAAGTCTGGAAACGCGGAAGTCAGCGCCCTGCACCATTATGTTCCGGATCTGCATCGCAGGATGCTGCTGGCTACCCTGTGGAACACCTACATCTGTATTAACGAAGCGCTGGCATTGACCCTGAGTGATTTTTCTCTGGTCCCGCCGCATCCATACCGCCAGTTGTTTACCCTCACAACGTTCCAGTAACCGGGCATGTTCATCATCAGTAACCCGTATCGTGAGCATCCTCTCTCGTTTCATCGGTATCATTACCCCCATGAACAGAAATCCCCCTTACACGGAGGCATCAGTGACCAAACAGGAAAAAACCGCCCTTAACATGGCCCGCTTTATCAGAAGCCAGACATTAACGCTTCTGGAGAAACTCAACGAGCTGGACGCGGATGAACAGGCAGACATCTGTGAATCGCTTCACGACCACGCTGATGAGCTTTACCGCAGCTGCCTCGCGCGTTTCGGTGATGACGGTGAAAACCTCTGACACATGCAGCTCCCGGAGACGGTCACAGCTTGTCTGTAAGCGGATGCCGGGAGCAGACAAGCCCGTCAGGGCGCGTCAGCGGGTGTTGGCGGGTGTCGGGGCGCAGCCATGACCCAGTCACGTAGCGATAGCGGAGTGTATACTGGCTTAACTATGCGGCATCAGAGCAGATTGTACTGAGAGTGCACCATATATGCGGTGTGAAATACCGCACAGATGCGTAAGGAGAAAATACCGCATCAGGCGCTCTTCCGCTTCCTCGCTCACTGACTCGCTGCGCTCGGTCGTTCGGCTGCGGCGAGCGGTATCAGCTCACTCAAAGGCGGTAATACGGTTATCCACAGAATCAGGGGATAACGCAGGAAAGAACATGTGAGCAAAAGGCCAGCAAAAGGCCAGGAACCGTAAAAAGGCCGCGTTGCTGGCGTTTTTCCATAGGCTCCGCCCCCCTGACGAGCATCACAAAAATCGACGCTCAAGTCAGAGGTGGCGAAACCCGACAGGACTATAAAGATACCAGGCGTTTCCCCCTGGAAGCTCCCTCGTGCGCTCTCCTGTTCCGACCCTGCCGCTTACCGGATACCTGTCCGCCTTTCTCCCTTCGGGAAGCGTGGCGCTTTCTCATAGCTCACGCTGTAGGTATCTCAGTTCGGTGTAGGTCGTTCGCTCCAAGCTGGGCTGTGTGCACGAACCCCCCGTTCAGCCCGACCGCTGCGCCTTATCCGGTAACTATCGTCTTGAGTCCAACCCGGTAAGACACGACTTATCGCCACTGGCAGCAGCCACTGGTAACAGGATTAGCAGAGCGAGGTATGTAGGCGGTGCTACAGAGTTCTTGAAGTGGTGGCCTAACTACGGCTACACTAGAAGGACAGTATTTGGTATCTGCGCTCTGCTGAAGCCAGTTACCTTCGGAAAAAGAGTTGGTAGCTCTTGATCCGGCAAACAAACCACCGCTGGTAGCGGTGGTTTTTTTGTTTGCAAGCAGCAGATTACGCGCAGAAAAAAAGGATCTCAAGAAGATCCTTTGATCTTTTCTACGGGGTCTGACGCTCAGTGGAACGAAAACTCACGTTAAGGGATTTTGGTCATGAACAATAAAACTGTCTGCTTACATAAACAGTAATACAAGGGGTGTTATGAGCCATATTCAACGGGAAACGTCTTGCTCTAGGCCGCGATTAAATTCCAACATGGATGCTGATTTATATGGGTATAAATGGGCTCGCGATAATGTCGGGCAATCAGGTGCGACAATCTATCGATTGTATGGGAAGCCCGATGCGCCAGAGTTGTTTCTGAAACATGGCAAAGGTAGCGTTGCCAATGATGTTACAGATGAGATGGTCAGACTAAACTGGCTGACGGAATTTATGCCTCTTCCGACCATCAAGCATTTTATCCGTACTCCTGATGATGCATGGTTACTCACCACTGCGATCCCCGGGAAAACAGCATTCCAGGTATTAGAAGAATATCCTGATTCAGGTGAAAATATTGTTGATGCGCTGGCAGTGTTCCTGCGCCGGTTGCATTCGATTCCTGTTTGTAATTGTCCTTTTAACAGCGATCGCGTATTTCGTCTCGCTCAGGCGCAATCACGAATGAATAACGGTTTGGTTGATGCGAGTGATTTTGATGACGAGCGTAATGGCTGGCCTGTTGAACAAGTCTGGAAAGAAATGCATAAACTTTTGCCATTCTCACCGGATTCAGTCGTCACTCATGGTGATTTCTCACTTGATAACCTTATTTTTGACGAGGGGAAATTAATAGGTTGTATTGATGTTGGACGAGTCGGAATCGCAGACCGATACCAGGATCTTGCCATCCTATGGAACTGCCTCGGTGAGTTTTCTCCTTCATTACAGAAACGGCTTTTTCAAAAATATGGTATTGATAATCCTGATATGAATAAATTGCAGTTTCATTTGATGCTCGATGAGTTTTTCTAAGAATTAATTCATGAGCGGATACATATTTGAATGTATTTAGAAAAATAAACAAATAGGGGTTCCGCGCACATTTCCCCGAAAAGTGCCACCTG

**sgRNA array sequence**

The P43 promoter is coloured purple and N20 is lighted yellow. sgRNA handle is coloured blue.

ATTTTACATTTTTAGAAATGGGCGTGAAAAAAAGCGCGCGATTATGTAAAATATAANNNNNNNNNNNNNNNNNNNNGTTTTAGAGCTAGAAATAGCAAGTTAAAATAAGGCTAGTCCGTTATCAACTTGAAAAAGTGGCACCGAGTCGGTGCGGTAATAAAAAAACACCTCCAAGCTGAGTGCGGGTATCAGCTTGGAGGTGCGTTTATTTTTTCAGCCGTATGACAAGGTCGGCATCAGGTGTGACAAATACGGTATGCTGGCTGTCATAGGTGACAAATCCGGGTTTTGCGCCGTTTGGCTTTTTCACATGTCTGATTTTTGTATAATCAACAGGCACGGAGCCGGAATCTTTCGCCTTGGAAAAATAAGCGGCGATCGTAGCTGCTTCCAATATGGATTGTTCATCGGGATCGCTGCTTTTAATCACAACGTGGGAATTTTACATTTTTAGAAATGGGCGTGAAAAAAAGCGCGCGATTATGTAAAATATAANNNNNNNNNNNNNNNNNNNNGTTTTAGAGCTAGAAATAGCAAGTTAAAATAAGGCTAGTCCGTTATCAACTTGAAAAAGTGGCACCGAGTCGGTGCGGTAATAAAAAAACACCTCCAAGCTGAGTGCGGGTATCAGCTTGGAGGTGCGTTTATTTTTTCAGCCGTATGACAAGGTCGGCATCAGGTGTGACAAATACGGTATGCTGGCTGTCATAGGTGACAAATCCGGGTTTTGCGCCGTTTGGCTTTTTCACATGTCTGATTTTTGTATAATCAACAGGCACGGAGCCGGAATCTTTCGCCTTGGAAAAATAAGCGGCGATCGTAGCTGCTTCCAATATGGATTGTTCATCGGGATCGCTGCTTTTAATCACAACGTGGGA

**mCherry-GHP integration plasmid (pET-mcherry-gfp) sequence**

The PamyQ’ promoter is coloured purple, The PspoVG promoter is coloured orange, gfp is lighted green and mcherry is lighted red. Homology arm regions are underlined. Cre-lox sequences are marked in gray.

TAAAGGTGACCCTTCCAAAAGGCTGAAGCTTGTTGAAAGCACGACTGATGCCCTTGGATACAAGCACTTTCGATATGCGCCTGTCGTTAACGGAGTGCCAATTAAAGATTCGCAAGTGATCGTTCACGTCGATAAATCCGATAATGTCTATGCGGTCAATGGTGAATTACACAATCAATCTGCTGCAAAAACAGATAACAGCCAAAAAGTCTCTTCTGAAAAAGCGCTGGCACTCGCTTTCAAACTCGAGATCGAGGCAAATCACCAGACGCTGTTTCTAACGGAGCGGCCAAAAACAGCAATAAAGCCGAATTAAAAGCGATAGAAACAAAAGACGGCAGCTATCGTCTTGCTTACGACGTGACGATTCGCTATGTCGAGCCTGAACCTGCAAACTGGGAAGTCTTAGTTGACGCCGAAACAGGCAGCATTTTAAAACAGCAAAATAAAGTAGAACATGCCGCCGCCACTGGAAGCGGAACAACGCTAAAGGGCGCAACTGTTCCTTTGAACATCTCTTATGAAGGCGGAAAATATGTTCTAAGAGATCTTTCAAAACCAACAGGCACCCAAATCATCACATATGATTTGCAAAACAGACAAAGCCGCCTTCCGGGCACGCTTGTCTCAAGCACAACGAAAACATTTACATCTTCATCACAGCGGGCAGCCGTTGACGCACACTATAACCTCGGTAAAGTGTACGATTATTTTTATTCAAACTTTAAACGAAACAGCTATGATAACAAAGGCAGTAAAATCGTTTCTTCCGTTCACTACGGCACTCAATACAATAACGCTGCATGGACAGGAGACCAGATGATTTACGGTGATGGCGACGGTTCATTCTTCTCTCCGCTTTCCGGCTCATTAGATGTGACAGCGCATGAAATGACACATGGCGTCACCCAAGAAACAGCCAACTTGATTTATGAAAATCAGCCAGGTGCATTAAACGAGTCTTTCTCTGACGTATTCGGGTATTTTAACGATACAGAAGACTGGCAAAAGCAGTCCACACAAAACATGCCCCAGCGGCAATCGGGCCTTATTCACAAGGGATTATCGTCAACAATATGTTTTACAGCTCAGGCCAAATCCCTTTGACTCCTTCAGGCGAAATGGTGAATGGCGATATTAAGGAGCAGACTCATCAAGTATTCAGCAATTTAAAGGCGGTTCTGGAAGAAGCGGGTGCTTCTTTTGAAACAGTTGTAAAAGCAACTGTATTTATCGCGGATATGGAACAGTTTGCGGAAGTAAACGAAGTGTACGGACAATATTTTGACACTCACAAACCGGCGAGATCTTGTGTTGAAGTCGCGAGACTCCCGAAGGATGCGTTAGTCGAGATCGAAGTTATTGCACTGGTGAAATAATAAGAAAAGTGATTCTGGGAGAGCCGGGATCACTTTTTTATTTACCTTATGCCCGAAATGAAAGCTTTATGACCTAATTGTGTAACTATATCCTATTTTTTCAAAAAATATTTTAAAAACGAGCAGGATTTCAGAAAAAATCGTGGAATTGATACACTAATGCTTTTATATAGGGAAAAGGTGGTGAACTACTATGGTTTCTAAAGGTGAAGAAGATAACATGGCTATCATCAAAGAATTTATGCGTTTCAAAGTTCACATGGAAGGTTCTGTTAACGGTCACGAATTTGAAATCGAAGGTGAAGGTGAAGGTCGTCCGTACGAAGGTACCCAGACCGCTAAACTGAAAGTTACCAAAGGTGGTCCGCTGCCGTTCGCTTGGGACATCCTGTCTCCGCAGTTCATGTACGGTTCTAAAGCGTACGTTAAACACCCGGCTGACATCCCGGACTACCTGAAACTGTCTTTCCCGGAAGGTTTCAAATGGGAACGTGTTATGAACTTCGAAGATGGTGGTGTTGTTACCGTTACCCAGGACTCTTCTCTGCAAGACGGTGAATTTATCTACAAAGTTAAACTGCGTGGTACCAACTTCCCGTCTGACGGTCCGGTTATGCAGAAAAAAACTATGGGTTGGGAAGCGAGCTCTGAACGTATGTACCCGGAAGATGGTGCTCTGAAAGGTGAAATCAAACAGCGTCTGAAACTGAAAGACGGTGGTCACTACGACGCTGAAGTTAAAACCACCTACAAAGCTAAAAAACCGGTTCAGCTGCCGGGTGCTTACAACGTTAACATCAAACTGGACATCACCTCTCACAACGAAGATTACACCATCGTTGAACAGTACGAACGTGCTGAAGGTCGTCACTCTACCGGTGGTATGGACGAACTGTACAAATAAATCTCCTGCAGGCATGCAAGCTTGAGTAGGACAAATCCGCCGAGCTTCGACGAGATTTTCAGGAGCTAAGGAAGCTAAAATGGAGAAAAAAATCACTGGATATACCACCGTTGATATATCCCAATGGCATCGGGCGGCGTTCTGTTTCTGCTTCGGTATGTGATTGTGAAGCTGGCTTACAGAAGAGCGGTAAAAGAAGAAATAAAAAAGAAATCATCTTTTTTGTTTGGAAAGCGAGGGAAGCGTTCACAGTTTCGGGCAGCTTTTTTTATAGGAACATTGATTTGTATTCACTCTGCCAAGTTGTTTTGATAGAGTGATTGTGATAATTTTAATGTAAGCGATAACAAAATTCTCCAGTCTTCACATCGGTTTGAAAGGAGGAAGCGGAAGAATGAAGTAAGAGGGATTTTTGACTCCGAAGTAAGTCTTCAAAAAATCAAATAAGGAGTGTCAAGAATGAGTAAAGGAGAAGAACTTTTCACTGGAGTTGTCCCAATTCTTGTTGAATTAGATGGTGATGTTAATGGGCACAAATTTTCTGTCAGTGGAGAGGGTGAAGGTGATGCAACATACGGAAAACTTACCCTTAAATTTATTTGCACTACTGGAAAACTACCTGTTCCATGGCCAACACTTGTCACTACTTTCGGTTATGGTGTTCAATGCTTTGCGAGATACCCAGATCATATGAAACAGCATGACTTTTTCAAGAGTGCCATGCCCGAAGGTTATGTACAGGAAAGAACTATATTTTTCAAAGATGACGGGAACTACAAGACACGTGCTGAAGTCAAGTTTGAAGGTGATACCCTTGTTAATAGAATCGAGTTAAAAGGTATTGATTTTAAAGAAGATGGAAACATTCTTGGACACAAATTGGAATACAACTATAACTCACACAATGTATACATCATGGCAGACAAACAAAAGAATGGAATCAAAGTTAACTTCAAAATTAGACACAACATTGAAGATGGAAGCGTTCAACTAGCAGACCATTATCAACAAAATACTCCAATTGGCGATGGCCCTGTCCTTTTACCAGACAACCATTACCTGTCCACACAATCTGCCCTTTCGAAAGATCCCAACGAAAAGAGAGACCACATGGTCCTTCTTGAGTTTGTAACAGCTGCTGGGATTACACATGGCATGGATGAACTATACAAATAAGAGCTCGGTACCCTCGAGGGATCCGAATTCAAGCTTGTCGACCTGCAGTCTAGACATCACCATCATCACCACTAATGCGGTAGTTTATCACAGTTAAATTGCTAACGCAGTCAGGCACCGTGTATGAAATCTAACAATGCGCTCATCGTCATCCTCGGCACCGTCACCCTGGATGCTGTAGGCATAGGCTTGGTTATGCCGGTACTGCCGGGTACCGTTCGTATAGCATACATTATACGAAGTTATTTCAACAAACGGGCCATATTGTTGTATAAGTGATGAAATACTGAATTTAAAACTTAGTTTATATGTGGTAAAATGTTTTAATCAAGTTTAGGAGGAATTAATTATGAAGTGTAATGAATAATGAATGTAACAGGGTTCAATTAAAAGAGGGAAGCGTATCATTAACCCTATAAACTACGTCTGCCCTCATTATTGGAGGGTGAAATGTGAATACATCCTATTCACAATCGAATTTACGACACAACCAAATTTTAATTTGGCTTTGCATTTTATCTTTTTTTAGCGTATTAAATGAAATGGTTTTGAACGTCTCATTACCTGATATTGCAAATGATTTTAATAAACCACCTGCGAGTACAAACTGGGTGAACACAGCCTTTATGTTAACCTTTTCCATTGGAACAGCTGTATATGGAAAGCTATCTGATCAATTAGGCATCAAAAGGTTACTCCTATTTGGAATTATAATAAATTGTTTCGGGTCGGTAATTGGGTTTGTTGGCCATTCTTTCTTTTCCTTACTTATTATGGCTCGTTTTATTCAAGGGGCTGGTGCAGCTGCATTTCCAGCACTCGTAATGGTTGTAGTTGCGCGCTATATTCCAAAGGAAAATAGGGGTAAAGCATTTGGTCTTATTGGATCGATAGTAGCCATGGGAGAAGGAGTCGGTCCAGCGATTGGTGGAATGATAGCCCATTATATTCATTGGTCCTATCTTCTACTCATTCCTATGATAACAATTATCACTGTTCCGTTTCTTATGAAATTATTAAAGAAAGAAGTAAGGATAAAAGGTCATTTTGATATCAAAGGAATTATACTAATGTCTGTAGGCATTGTATTTTTTATGTTGTTTACAACATCATATAGCATTTCTTTTCTTATCGTTAGCGTGCTGTCATTCCTGATATTTGTAAAACATATCAGGAAAGTAACAGATCCTTTTGTTGATCCCGGATTAGGGAAAAATATACCTTTTATGATTGGAGTTCTTTGTGGGGGAATTATATTTGGAACAGTAGCAGGGTTTGTCTCTATGGTTCCTTATATGATGAAAGATGTTCACCAGCTAAGTACTGCCGAAATCGGAAGTGTAATTATTTTCCCTGGAACAATGAGTGTCATTATTTTCGGCTACATTGGTGGGATACTTGTTGATAGAAGAGGTCCTTTATACGTGTTAAACATCGGAGTTACATTTCTTTCTGTTAGCTTTTTAACTGCTTCCTTTCTTTTAGAAACAACATCATGGTTCATGACAATTATAATCGTATTTGTTTTAGGTGGGCTTTCGTTCACCAAAACAGTTATATCAACAATTGTTTCAAGTAGCTTGAAACAGCAGGAAGCTGGTGCTGGAATGAGTTTGCTTAACTTTACCAGCTTTTTATCAGAGGGAACAGGTATTGCAATTGTAGGTGGTTTATTATCCATACCCTTACTTGATCAAAGGTTGTTACCTATGGAAGTTGATCAGTCAACTTATCTGTATAGTAATTTGTTATTACTTTTTTCAGGAATCATTGTCATTAGTTGGCTGGTTACCTTGAATGTATATAAACATTCTCAAAGGGATTTCTAAATCGTTAAGGGATCAACTTTGGGAGAGAGTTCAAAATTGATCCTTTTTTTATAACAGGAATTCCCGGGGATCCGTCGACCTGCAGATCTCTAGAAGCTTATAACTTCGTATAGCATACATTATACGAACGGTATGAAGACATTACGGTCAGCCAGCCTGCTCTTCGCAGCCTGTCCAACCCTACAAAATACAACCAGCCTGACAATTACGCCAATTACCGAAACCTTCCAAACACAGATGAAGGCGATTATGGCGGTGTACACACAAACAGCGGAATTCCAAACAAAGCCGCTTACAACACCATCACAAAACTTGGTGTATCTAAATCACAGCAAATCTATTACCGTGCGTTAACAACGTACCTCACGCCTTCTTCCACGTTCAAAGATGCCAAGGCAGCTCTCATTCAGTCTGCCCGTGACCTCTACGGCTCAACTGATGCCGCTAAAGTTGAAGCAGCCTGGAATGCTGTTGGATTGTAATATTAGGAAAAGCCTGAGATCCCTCAGGCTTTTATTGTTACATATCTTGATTTCTCTCTCAGCTGAAACGACGAAAAGATGCTGCCATGAGACAGAAAACCGCTCCTGATTTGCATAAAGAGGGATGCAGCCGCAAGTGCGCATTTTATAAAAGCTAATGATTCAGTCCACATAATTGATAGACGAATTCTGCTACAGGTCACGTGGCTATGTGAAGGATCGCGCGTCCAGTTAAGAGCAAAAACATTGACAAAAAAATTTATTTATGCTAAAATTTACTATTAATATATTTGTATGTATAATAAGATTCTCCTGGCCAGGGGAATCTTATTTTTTGTGGAGGATCATTTCATGAGGAAAAATGAGTCCAGCTTAACGTCTCTAATTTCAGCTTTTGCCCGTGCATATCACAGCCGATATGACACACCTCTTATTTTTGATGATTTTATCGCAAAAGATCTCATTAACGAAAAAGAGTTTATCGACATCAGTAAAAATATGATTCAAGAAATATCGTTTTTCAACAAAGAGATCGCCGAACGTCTTCAAAATGATCCTGAAAAAATATTAAAATGGGTTGCACAAATCCAGCTGTCTCCAACGCCCCTAGCACGTGCTTCTTATTGTGAAAAAGTCTTGCACAACGAATTACCTATTTCACTTTTTGCATTCTACAAACTGCATAACTATTATGGATCACTCAAAAAATCTCCACCTTTAAACCCTTGCCAATTTTTATTTTGTCCGTTTTGTCTAGCTTACCGAAAGCCAGACTCAGCAAGAATAAAATTTTTATTGTCTTTCGGTTTTCTAGTGTAACGGACAAAACCACTCAAAATAAAAAAGATACAAGAGAGGTCTCTCGTATCTTTTATTCAGCAATCGCGCCCGATTGCTGAACAGATTAATAATAGATTTTAGCTTTTTATTTGTTGAAAAAAGCTAATCAAATTGTTGTCGGGATCAATTACTGCAAAGTCTCGTTCATCCCACCACTGATCTTTTAATGATGTATTGGGGTGCAAAATGCCCAAAGGCTTAATATGTTGATATAATTCATCAATTCCCTCTACTTCAATGCGGCAACTAGCAGTACCAGCAATAAACGACTCCGCACCTGTACAAACCGGTGAATCATTACTACGAGAGCGCCAGCCTTCATCACTTGCCTCCCATAGATGAATCCGAACCTCATTACACATTAGAACTGCGAATCCATCTTCATGGTGAACCAAAGTGAAACCTAGTTTATCGCAATAAAAACCTATACTCTTTTTAATATCCCCGACTGGCAATGCCGGGATAGACTGTAACATTCTCACGCATAAAATCCCCTTTCATTTTCTAATGTAAATCTATTACCTTATTATTAATTCAATTCGCTCATAATTAATCCTTTTTCTTATTACGCAAAATGGCCCGATTTAAGCACACCCTTTATTCCGTTAATGCGCCATGACAGCCATGATAATTACTAATACTAGGAGAAGTTAATAAATACGAGCAAAAGGCCAGCAAAAGGCCAGGAACCGTAAAAAGGCCGCGTTGCTGGCGTTTTTCCATAGGCTCCGCCCCCCTGACGAGCATCACAAAAATCGACGCTCAAGTCAGAGGTGGCGAAACCCGACAGGACTATAAAGATACCAGGCGTTTCCCCCTGGAAGCTCCCTCGTGCGCTCTCCTGTTCCGACCCTGCCGCTTACCGGATACCTGTCCGCCTTTCTCCCTTCGGGAAGCGTGGCGCTTTCTCATAGCTCACGCTGTAGGTATCTCAGTTCGGTGTAGGTCGTTCGCTCCAAGCTGGGCTGTGTGCACGAACCCCCCGTTCAGCCCGACCGCTGCGCCTTATCCGGTAACTATCGTCTTGAGTCCAACCCGGTAAGACACGACTTATCGCCACTGGCAGCAGCCACTGGTAACAGGATTAGCAGAGCGAGGTATGTAGGCGGTGCTACAGAGTTCTTGAAGTGGTGGCCTAACTACGGCTACACTAGAAGAACAGTATTTGGTATCTGCGCTCTGCTGAAGCCAGTTACCTTCGGAAAAAGAGTTGGTAGCTCTTGATCCGGCAAACAAACCACCGCTGGTAGCGGTGGTTTTTTTGTTTGCAAGCAGCAGATTACGCGCAGAAAAAAAGGATCTCAAGAAGATCCTTTGATCTTTTCTACGGGGTCTGACGCTCAGTGGAACGAAAACTCACGTTAAGGGATTTTGGTCATGAGATTATCAAAAAGGATCTTCACCTAGATCCTTTTAAATTAAAAATGAAGTTTTAAATCAATCTAAAGTATATATGAGTAAACTTGGTCTGACAGTTACCAATGCTTAATCAGTGAGGCACCTATCTCAGCGATCTGTCTATTTCGTTCATCCATAGTTGCCTGACTCCCCGTCGTGTAGATAACTACGATACGGGAGGGCTTACCATCTGGCCCCAGTGCTGCAATGATACCGCGAGACCCACGCTCACCGGCTCCAGATTTATCAGCAATAAACCAGCCAGCCGGAAGGGCCGAGCGCAGAAGTGGTCCTGCAACTTTATCCGCCTCCATCCAGTCTATTAATTGTTGCCGGGAAGCTAGAGTAAGTAGTTCGCCAGTTAATAGTTTGCGCAACGTTGTTGCCATTGCTACAGGCATCGTGGTGTCACGCTCGTCGTTTGGTATGGCTTCATTCAGCTCCGGTTCCCAACGATCAAGGCGAGTTACATGATCCCCCATGTTGTGCAAAAAAGCGGTTAGCTCCTTCGGTCCTCCGATCGTTGTCAGAAGTAAGTTGGCCGCAGTGTTATCACTCATGGTTATGGCAGCACTGCATAATTCTCTTACTGTCATGCCATCCGTAAGATGCTTTTCTGTGACTGGTGAGTACTCAACCAAGTCATTCTGAGAATAGTGTATGCGGCGACCGAGTTGCTCTTGCCCGGCGTCAATACGGGATAATACCGCGCCACATAGCAGAACTTTAAAAGTGCTCATCATTGGAAAACGTTCTTCGGGGCGAAAACTCTCAAGGATCTTACCGCTGTTGAGATCCAGTTCGATGTAACCCACTCGTGCACCCAACTGATCTTCAGCATCTTTTACTTTCACCAGCGTTTCTGGGTGAGCAAAAACAGGAAGGCAAAATGCCGCAAAAAAGGGAATAAGGGCGACACGGAAATGTTGAATACTCATACTCTTCCTTTTTCAATATTATTGAAGCATTTATCAGGGTTATTGTCTCATGAGCGGATACATATTTGAATGTATTTAGAAAAATAAACAAATAGGGGTTCCGCGCACATTTCCCCGAAAAGTGCCACCTGACGTCTAAGAAACCATTATTATCATGACATTAACCTATAAAAATAGGCGTATCACGAGGCCCTTTCGTCTCGCGCGTTTCGGTGATGACGGTGAAAACCTCTGACAGTAACCAACATGATTAACAATTATTAGAGGTCATCGTTCAAAATGGTATGCGTTTTGACACATCCACTATATATCCGTGTCGTTCTGTCCACTCCTGAATCCCATTCCAGAAATTCTCTAGCGATTCCAGAAGTTTCTCAGAGTCGGAAAGTTGACCAGACATTACGAACTGGCACAGATGGTCATAACCTGAAGGAAGATCTGATTGCTTAACTGCTTCAGTTAAGACCGAAGCGCTCGTCGTATAACAGATGCGATGATGCAGACCAATCAACATGGCACCTGCCATTGCTACCTGTACAGTCAAGGATGGTAGAAATGTTGTCGGTCCTTGCACACGAATATTACGCCATTTGCCTGCATATTCAAACAGCTCTTCTACGATAAGGGCACAAATCGCATCGTGGAACGTTTGGGCTTCTACCGATTTAGCAGTTTGATACACTTTCTCTAAGTATCCACCTGAATCATAAATCGGCAAAATAGAGAAAAATTGACCATGTGTAAGCGGCCAATCTGATTCCACCTGAGATGCATAATCTAGTAGAATCTCTTCGCTATCAAAATTCACTTCCACCTTCCACTCACCGGTTGTCCATTCATGGCTGAACTCTGCTTCCTCTGTTGACATGACACACATCATCTCAATATCCGAATAGGGCCCATCAGTCTGACGACCAAGAGAGCCATAAACACCAATAGCCTTAACATCATCCCCATATTTATCCAATATTCGTTCCTTAATTTCATGAACAATCTTCATTCTTTCTTCTCTAGTCATTATTATTGGTCCATTCACTATTCTCATTCCCTTTTCAGATAATTTTAGATTTGCTTTTCTAAATAAGAATATTTGGAGAGCACCGTTCTTATTCAGCTATTAATAACTCGTCTTCCTAAGCATCCTTCAATCCTTTTAATAACAATTATAGCATCTAATCTTCAACAAACTGGCCCGTTTGTTGAACTACTCTTTAATAAAATAATTTTTCCGTTCCCAATTCCACATTGCAATAATAGAAAATCCATCTTCATCGGCTTTTTCGTCATCATCTGTATGAATCAAATCGCCTTCTTCTGTGTCATCAAGGTTTAATTTTTTATGTATTTCTTTTAACAAACCACCATAGGAGATTAACCTTTTACGGTGTAAACCTTCCTCCAAATCAGACAAACGTTTCAAATTCTTTTCTTCATCATCGGTCATAAAATCCGTATCCTTTACAGGATATTTTGCAGTTTCGTCAATTGCCGATTGTATATCCGATTTATATTTATTTTTCGGTCGAATCATTTGAACTTTTACATTTGGATCATAGTCTAATTTCATTGCCTTTTTCCAAAATTGAATCCATTGTTTTTGATTCACGTAGTTTTCTGTATTCTTAAAATAAGTTGGTTCCACACATACCAATACATGCATGTGCTGATTATAAGAATTATCTTTATTATTTATTGTCACTTCCGTTGCACGCATAAAACCAACAAGATTTTTATTAATTTTTTTATATTGCATCATTCGGCGAAATCCTTGAGCCATATCTGACAAACTCTTATTTAATTCTTCGCCATCATAAACATTTTTAACTGTTAATGTGAGAAACAACCAACGAACTGTTGGCTTTTGTTTAATAACTTCAGCAACAACCTTTTGTGACTGAATGCCATGTTTCATTGCTCTCCTCCAGTTGCACATTGGACAAAGCCTGGATTTACAAAACCACACTCGATACAACTTTCTTTCGCCTGTTTCACGATTTTGTTTATACTCTAATATTTCAGCACAATCTTTTACTCTTTCAGCCTTTTTAAATTCAAGAATATGCAGAAGTTCAAAGTAATCAACATTAGCGATTTTCTTTTCTCTCCATGGTCTCACTTTTCCACTTTTTGTCTTGTCCACTAAAACCCTTGATTTTTCATCTGAATAAATGCTACTATTAGGACACATAATATTAAAAGAAACCCCCATCTATTTAGTTATTTGTTTAGTCACTTATAACTTTAACAGATGGGGTTTTTCTGTGCAACCAATTTTAAGGGTTTTCAATACTTTAAAACACATACATACCAACACTTCAACGCACCTTTCAGCAACTAAAATAAAAATGACGTTATTTCTATATGTATCAAGATAAGAAAGAACAAGTTCAAAACCATCAAAAAAAGACACCTTTTCAGGTGCTTTTTTTATTTTATAAACTCATTCCCTGATCTCGACTTCGTTCTTTTTTTACCTCTCGGTTATGAGTTAGTTCAAATTCGTTCTTTTTAGGTTCTAAATCGTGTTTTTCTTGGAATTGTGCTGTTTTATCCTTTACCTTGTCTACAAACCCCTTAAAAACGTTTTTAAAGGCTTTTAAGC
